# High monocytic MDSC signature predicts multi-drug resistance and cancer relapse in non-Hodgkin lymphoma patients treated with R-CHOP

**DOI:** 10.3389/fimmu.2023.1303959

**Published:** 2024-01-18

**Authors:** Sukanya Dhar, Mohona Chakravarti, Nilanjan Ganguly, Akata Saha, Shayani Dasgupta, Saurav Bera, Anirban Sarkar, Kamalika Roy, Juhina Das, Avishek Bhuniya, Sarbari Ghosh, Madhurima Sarkar, Srabanti Hajra, Saptak Banerjee, Chiranjib Pal, Bhaskar Saha, Kalyan Kusum Mukherjee, Rathindranath Baral, Anamika Bose

**Affiliations:** ^1^ Department of Immunoregulation and Immunodiagnostics, Chittaranjan National Cancer Institute, Kolkata, India; ^2^ Cellular Immunology and Experimental Therapeutics Laboratory, Department of Zoology, West Bengal State University, Barasat, India; ^3^ Department of Pathology, Chittaranjan National Cancer Institute, Kolkata, India; ^4^ Department of Pathogenesis and Cell Responses, National Centre for Cell Science, Pune, Maharashtra, India; ^5^ Department of Medical Oncology, Chittaranjan National Cancer Institute, Kolkata, India

**Keywords:** cancer recurrence, IL-6/IL-10/IL-1β, multi-drug resistance, myeloid derived suppressor cells, non-Hodgkin lymphoma, therapy resistance, tumor microenvironment

## Abstract

**Introduction:**

Non-Hodgkin Lymphoma (NHL) is a heterogeneous lymphoproliferative malignancy with B cell origin. Combinatorial treatment of rituximab, cyclophsphamide, hydroxydaunorubicin, oncovin, prednisone (R-CHOP) is the standard treatment regimen for NHL, yielding a complete remission (CR) rate of 40-50%. Unfortunately, considerable patients undergo relapse after CR or initial treatment, resulting in poor clinical implications. Patient’s response to chemotherapy varies widely from static disease to cancer recurrence and later is primarily associated with the development of multi-drug resistance (MDR). The immunosuppressive cells within the tumor microenvironment (TME) have become a crucial target for improving the therapy efficacy. However, a better understanding of their involvement is needed for distinctive response of NHL patients after receiving chemotherapy to design more effective front-line treatment algorithms based on reliable predictive biomarkers.

**Methods:**

Peripheral blood from 61 CD20^+^ NHL patients before and after chemotherapy was utilized for immunophenotyping by flow-cytometry at different phases of treatment. *In-vivo* and *in-vitro* doxorubicin (Dox) resistance models were developed with murine Dalton’s lymphoma and Jurkat/Raji cell-lines respectively and impact of responsible immune cells on generation of drug resistance was studied by RT-PCR, flow-cytometry and colorimetric assays. Gene silencing, ChIP and western blot were performed to explore the involved signaling pathways.

**Results:**

We observed a strong positive correlation between elevated level of CD33^+^CD11b^+^CD14^+^CD15^-^ monocytic MDSCs (M-MDSC) and MDR in NHL relapse cohorts. We executed the role of M-MDSCs in fostering drug resistance phenomenon in doxorubicin-resistant cancer cells in both *in-vitro, in-vivo* models. Moreover, *in-vitro* supplementation of MDSCs in murine and human lymphoma culture augments early expression of MDR phenotypes than culture without MDSCs, correlated well with *in-vitro* drug efflux and tumor progression. We found that MDSC secreted cytokines IL-6, IL-10, IL-1β are the dominant factors elevating MDR expression in cancer cells, neutralization of MDSC secreted IL-6, IL-10, IL-1β reversed the MDR trait. Moreover, we identified MDSC secreted IL-6/IL-10/IL-1β induced STAT1/STAT3/NF-κβ signaling axis as a targeted cascade to promote early drug resistance in cancer cells.

**Conclusion:**

Our data suggests that screening patients for high titre of M-MDSCs might be considered as a new potential biomarker and treatment modality in overcoming chemo-resistance in NHL patients.

## 1 Introduction

Non-Hodgkin lymphoma (NHL) is the most prevalent hematologic malignancy across the globe (1) comprising 4.3% of total cancer diagnosed with a below 72% five years survival rate (2). Nearly 25% of NHL cases arise in extranodal locations and most of them are seen involving both nodal and extranodal sites (3). The majority of NHL originates from B cells with about 10% arising from T cells or natural killer (NK) cells (4, 5). Between two prognostic groups - aggressive and indolent lymphomas; the former accounts for 60% of reported incidences. Among aggressive lymphomas, diffuse large B-cell lymphoma (DLBCL) is the most common high-grade and aggressive B-NHL. Slow-growing or indolent subtypes represent about 40% of all NHL cases and follicular lymphoma (FL) is the most common subtype of indolent NHL (2). The anthracycline based chemotherapy regimen, termed R-CHOP represents the new-standard treatment for NHL and composed of cyclophosphamide, hydroxydaunorubicin, oncovin, prednisone along with rituximab-(monoclonal antibody to CD20) (6, 7) . Most NHL patients initially respond to chemotherapy yielding complete response rate of 40–50% (8, 9). Unfortunately, a substantial population of patients undergo relapse, resulting in poor clinical ramifications (9). The onset of NHL relapse evolves from several months (early relapse) to years (late relapse) after the initial remission. However, the majority of relapse occurs within two years of initial treatment. Patients with relapsed NHL can be divided into two broad categories: patients, who relapse even after complete remission of cancer after chemotherapy and in another group, where patients who do not experience a complete disappearance of their cancer after chemotherapy.

Despite considerable advancement in therapeutic concepts and techniques, disease relapse with limited response rate remains a major challenge for successful clinical management. Patient’s response to chemotherapy varies widely from static disease to cancer recurrence and the latter is primarily associated with generation of multi-drug-resistance (MDR) phenotypes, that ultimately paralyzes immune surveillance (10). Resistance to different chemotherapeutic regimen involves intrinsic and extrinsic mechanisms and those are determined by pre-existing genetic and/or epigenetic properties of malignant cells including enhanced drug efflux pumps (i.e. P-glycoprotein; MDR1; MRP1), blunted apoptotic signaling, increased metabolic activities, loss of specific oncogenes, gain of stem cell plasticity, strengthen DNA damage repair machinery, tumor heterogeneity within tumor microenvironment (TME) (11, 12). However, the mechanisms crucial to differential responses and outcomes to standard chemotherapeutic regimens in NHL patients particularly with DLBCL subtypes are yet be elucidated.

An important outlook associated with disease recurrence in NHL patients after chemotherapy is the immune-suppressive effects generated particularly within TME (13, 14) . Several reports suggested the presence of immune suppressor cells and their secreted factors influenced response to therapy and patients’ prognosis (15, 16). A censorious step in disease progression is immune evasion and suppression of host immune system by T regulatory cells (Treg), myeloid derived suppressor cells (MDSCs), M2 macrophages that paralyzes the host immune surveillance. Among these, a heterogeneous population of immature myeloid cells or MDSCs recognised as a major obstacle for anti-tumor immunity. In mice, MDSCs were classically identified as CD11b^+^Gr1^+^ cells and divided into two main subgroups with different suppression activities: polymorphonuclear or granulocytic MDSCs (PMN-MDSCs/G-MDSCs- CD11b^+^Ly6G^+^Ly6C^low^ cells) and monocytic MDSCs (M-MDSCs - CD11b^+^Ly6C^+^Ly6G^−^ cells) (16). Human M-MDSCs are defined as Lin^-^HLA-DR^-/low^CD11b^+^CD33^+^CD14^+^CD15^-^ and PMN-MDSCs/G-MDSCs as Lin^-^HLA-DR^-^CD11b^+^CD33^+^CD14^-^CD15^+^ or Lin^-^CD11b^+^CD14^-^CD66b^+^ (17). Immense plasticity and functional diversity of MDSCs create difficulty in targeting the responsible MDSC subsets and designing therapies to re-educate the immune paralyzing mechanism in context of chemotherapy management (18, 19). However, involvement of MDSC subtypes in modulation of cancer cells still remains unexplored in NHL patients.

In the current study, we observed an enhanced population of monocytic MDSCs (M-MDSC) within non-responder NHL patients including DLBCL, which follows a strong positive correlation with poor prognosis. *In-vitro* and *in-vivo* studies also suggested that M-MDSCs have a potential to generate early drug resistance niche, creating a major hurdles for relapsed and refractory NHL patients. Moreover, MDSC-secreted IL-6, IL-10, IL-1β appeared as dominant constituent in regulating expression of multidrug-resistance pump by modulating STAT1, STAT3 and NF-κβ signalling.

## 2 Methods

### 2.1 Reagents and antibodies

IMDM, RPMI-1640, and fetal bovine serum (FBS) were procured from Hi-Media (Mumbai, India). Anti-human CD33^+^ biotinylated antibody was purchased from BioLegend (San Diego, CA, USA). Anti-mouse/human fluorescence conjugated antibodies, purified monoclonal antibodies, reagents, like, Pgp (Invitrogen, Massachusetts, USA); MRP1 (Santa Cruz, Dallas, Texas, USA); CD4, CD8 (BD Pharmingen, San Diego, CA, USA); CD33, CD11b, CD14, CD163, CD206 (BioLegend, San Diego, CA, USA); CD25, FoxP3 (eBioscience, Fremont, CA, USA); p-STAT3 (Tyr 705) (BioLegend, San Diego, CA, USA); p-STAT3 (Ser 727) (Santa Cruz, Dallas, Texas, USA); STAT1 (BD Biosciences, San Jose, CA); p50 (Santa Cruz, Dallas, Texas, USA); p65 (Abcam, Cambridge, UK); p-IKB (R & D, Minneapolis, Canada, USA); Recombinant IL-6, IL-10, IL-1β (BD Pharmingen, San Diego, CA, USA); fluorescence conjugated secondary antibodies (Abcam, Cambridge, UK); Streptavidin Particles Plus-DM, Antibodies for ELISA (BD Biosciences, San Jose, CA) were procured from different vendors as indicated in parentheses. Heparin, β-mercaptoethanol, doxorubicin, aspirin, PMA, ionomycin, rhodamine 123 were purchased from Sigma-Aldrich (St. Louis, Missouri, USA). Opti-MEM was procured from Invitrogen (Carlsbad, CA, USA). RT-PCR primers were designed and procured from Eurofins, Bangalore, India. Trizol reagent for RNA isolation and Revert Aid™ cDNA synthesis kit were procured from Invitrogen (Carlsbad, CA, USA) and Fermentas (Waltham, MA, USA), respectively. STAT3 and STAT1 siRNAs were synthesized using Silencer^®^ Si-RNA construction kit (Life Technologies, USA) as per manufacturer’s protocol.

### 2.2 NHL patients’ blood

All blood samples were collected from patients as well as healthy individuals from Chittaranjan National Cancer Institute (CNCI), Kolkata, India following approval from Institutional Ethical Committee (IEC Ref: CNCI-IEC-RB-2020-3) after obtaining informed consent from every individual. Heparinized blood samples (5ml) of both pre- and post-chemotherapy patients (pre-chemotherapy, n=10; post-chemotherapy, n=51) were collected under supervision of medical oncologist. Blood from age and sex matched healthy individuals were used as control. Peripheral blood mononuclear cells (PBMCs) were isolated from blood by density gradient centrifugation using Lymphocyte Separation Media (LSM) (Hi-Media; Mumbai, India).

Information regarding patient’s history, course of treatment were collected from hospital record and tabulated in **Additional File 1, Supplementary Table 1**.

Based on differential response to chemotherapy, patients were classified into two groups:

1. Responder
(a) Disease Free: No relapse within 2 years or more than 2 years following initiation of treatment.
(b) Auto-regression: Regression of tumor spontaneously.
2. Non-responder
(a) Relapse/Recurrence: Return of the disease within a period of time (within 6 months or early relapse, after 2 years or late relapse).
(b) Persisting or Static: No further progression or improvement.

### 2.3 Human tumors

Biopsy tissue samples of both pre- and post-chemotherapy NHL patients (n=8 for pre-chemotherapy: n=24 for post-chemotherapy non-responder) were collected from Pathology department of CNCI, Kolkata, India. The tissues were collected aseptically and marker study was done for further analysis.

### 2.4 Mice and tumors

Wild-type (Wt) female Swiss Albino mice (age: 4 - 6 weeks; average body weight of 18-22 g) were obtained from the animal facility of CNCI, Kolkata. Autoclaved dry pellet diet (Epic Laboratory, West Bengal Government, Kalyani, India) and water were given *ad libitum*. Maintenance and treatment of animals were conducted according to the guidelines established by the Institutional Animal Care and Ethics Committee, CNCI, Kolkata (Approval No: IAEC-1774/RB-16/2017/3).

DL (Dalton’s Lymphoma), a murine transplantable T-cell lymphoma, were maintained in laboratory in Swiss albino mice through serial intraperitoneal (i.p) inoculation of tumor cells (1x10^5^ DL cells/mice) in ascitic form at an interval of 9–10 days under aseptic conditions. DL cells were tested for viability using Trypan blue dye exclusion method.

### 2.5 *In vivo* drug (Dox) resistance model in Dalton’s Lymphoma

A validated protocol was established to develop drug (Doxorubicin or Dox) resistant in *in-vivo* mice model with continuous exposure to Dox. For ascitic drug resistance model, 1x10^5^ DL cells were inoculated i.p in Swiss albino mice. After 24 hrs of tumor inoculation, mice were treated with Dox (2mg/kg/b.w.) for 10 days. These first sets of mice with Dox treatment denoted as Passage 1 or P1. After completion of drug treatment, tumor cells were taken from P1 mice and inoculated into another set of mice. The drug treatment was same as earlier group (like P1) and maintained for 10 days and this was denoted as passage 2 or P2. These passages were maintained upto 12 generations or P12 passages. The mice without drug treatment were denoted as Control (C). The passage cells were divided into three groups: early passage (EP), middle passage (MP), late passage (LP) for further analysis.

### 2.6 Cell lines and culture

Jurkat and Raji cell lines were obtained from National Centre for Cell Science (NCCS), Pune, India. Cell line authentication via STR profiling was conducted by cell repository. Cells were propagated in IMDM (for Jurkat), RPMI-1640 (for Raji) media, supplemented with 10% heat-inactivated FBS, 2 mM Glutamine-Penicillin-Streptomycin solution (Hi-Media, Mumbai, India). Cells were maintained in a humidified incubator at 37°C with supply of 5% CO_2_. Sub-culturing of Jurkat and Raji cells in suspension were performed when they reached around 70% confluency.

### 2.7 MTT assay for cell proliferation

Cells were seeded in 96-well plates at a cell density of 1x10^5^ cells/well for Jurkat and 5x10^4^ cells/well for Raji cell lines and exposed to Dox (starting from concentration 1 nM to 10 nM) for 48 hrs (Jurkat) and 24 hrs (Raji) cell lines respectively. On the scheduled day, 20 µl of 5 mg/ml MTT (Merck, USA) reagent was added to each well and incubated for 3 hrs. Next, the solution was removed and 100 µl of dimethyl sulfoxide (DMSO) was added to the wells. Then, the plate was read at 575 nm by using the microplate reader SpectraMax i3x (Molecular Devices, San Jose, USA). Data was accounted via SoftMax Pro 7.1 software. Cell viability was expressed in terms of percentage (%). IC_50_ value of Dox on studied cells was evaluated and expressed in molar concentration.

### 2.8 *In-vitro* Dox resistance model

To induce the doxorubicin-resistant phenotype (Dox-res), Jurkat and Raji cells were grown in IMDM and RPMI-1640 media respectively containing 10% FBS and exposed to IC_50_ dose of Dox as evaluated for each cell line. At the beginning of the experiment, counted 1x10^5^ cells were seeded in each 6 well of cell culture plates and after 48 hrs, the first sample without Dox treatment was harvested; this sample was named as initial passage (P0). Next, P0 cells were harvested and counted in a Neubauer chamber and required number of cells (determined by trypan blue dye exclusion assay) were seeded in a new 6 well cell culture plate and cultured immediately for 48 hrs with IC_50_ dose of Dox. After 48 hrs, the cells were harvested as P1 passage. This process was repeated with the fixed cell number and drug dose. Cells were considered chemo-resistant when exposure to IC_50_ dose of Dox, did not induce cellular apoptosis. The repeated dose of Dox developed chemo resistance, thus reluctant to cell death. In this study, cells became resistant to Dox at the end of 12^th^ passage (P12). Throughout the generation of Dox resistant passages, every sample of passages (P0 to P12) were collected. The same procedure was performed on untreated cells up to 12^th^ consecutive passages (control group) to identify possible alterations related to cell passages. The passage cells were divided into three groups: early passage (EP), middle passage (MP), late passage (LP) for further analysis.

### 2.9 Tumor-conditioned media

To obtain tumor-conditioned media (TCM), approximately 1× 10^5^ cancer cells (Jurkat/Raji) were cultured at 37°C in 5% CO_2_ and allowed to grow upto 70% confluency. After reaching the cells over 70% confluency, cells were centrifuged at 2,000 rpm for 30 mins, and culture supernatant (TCM) was kept in aliquots at −80°C (for single use) after filtration with 0.22 μm membrane filter (Millipore, Darmstadt, Germany). Protein content of the supernatant was determined by Bradford assay as described (20).

### 2.10 PBMC isolation and tumor conditioning

Blood (5 ml) from healthy donors was collected on heparinized collection tube, gently layered on 2.5 ml LSM and allowed for 30 mins of density gradient centrifugation at 1500 rpm. After centrifugation, the yielded white buffy coat having PBMCs were carefully pipetted out and washed with PBS to generate purified PBMCs.

For tumor conditioning, PBMCs (1×10^5^ cells/ml) were exposed to TCM at 1:5 ratio of RPMI-1640 in 6 well tissue culture treated plates. Following 72 hrs of culture, PBMCs were collected to get the desired degree of conditioning. Protein content of the supernatant was determined by Bradford assay as described.

### 2.11 *In vitro* MDSC generation

Heparinized blood was collected from healthy individuals to isolate PBMC by the method as described earlier. Briefly, PBMCs (1×10^5^ cells/ml) were cultured in complete RPMI-1640 medium in presence of TCM in 1:5 ratio in 6 well tissue culture treated plates. After 72 hrs, tumor conditioned PBMCs were harvested, centrifuged, collected and incubated further with anti-CD33 magnetic microbeads and CD33^+^ cells were sorted by BD iMAG sorter as per manufacturer’s instruction. Purity of isolated cell population was checked by flow-cytometry and cells with greater than 90% purity was used further. Cells were morphologically examined and viability of isolated cells was confirmed using trypan blue dye exclusion assay. To obtain culture supernatant of MDSCs, approximately 1×10^5^ MDSCs/ml were cultured in RPMI-1640 medium at 37°C in 5% CO_2_ for 48 hrs. Exhausted media was collected and centrifuged at 2,000 rpm for 30 mins, and culture supernatant was kept in aliquots at -80°C (for single use) after filtration as mentioned before. Protein content of the supernatant was determined by Bradford assay.

### 2.12 Co-culture and Transwell assay

For co-culture or contact-dependent (CD) assay, the purified CD33^+^ MDSCs were co-cultured *in-vitro* with Dox generated passages (early passage or EP, late passage or LP) of human Jurkat/Raji cells at 1:1 ratio (MDSCs: Cancer cells) for 48 hrs. Cancer cells without MDSCs were kept as control. After incubation period, CD33^+^ cells were sorted from mixture of cells and the remnant cancer cells were centrifuged to collect pellet which was further utilized for downstream signalling assays.

For transwell or contact-independent (CI) assay, purified CD33^+^cells were cultured with EP, LP of human cell line Jurkat/Raji cells at 1:1 ratio for 48 hrs in a contact independent manner. Both the target cells were separated physically by 0.4 µM transwell membrane (Hi-Media, Mumbai, India). Cancer cells were grown below the membrane, while CD33^+^ cells were laid into upper compartment of transwell inserts and incubated for indicated time periods in a humidified chamber at 37°C.

### 2.13 Total RNA isolation, cDNA synthesis and RT-PCR

Total RNA was isolated using the Trizol (Invitrogen, Camarillo, CA, USA) according to the manufacturer’s protocol. All RNA preparation and handling procedures were performed in a laminar air-flow hood under RNase-free conditions. Isolated RNA was dissolved in nuclease free water and stored at -80°C for further processing. The RNA concentration was evaluated by absorbance readings at 260 nm using a NanoDrop spectrophotometer (Thermo Scientific, Fremont, CA, USA). Random hexamers were used to generate corresponding cDNA (First Strand cDNA Synthesis Kit; Fermentas, Hanover, MD, USA). RT-PCR primers were prepared by Eurofins (Luxembourg, Germany) and listed in **Additional File 2, Supplementary Table 2**. Amplification of the target genes was performed using Go Taq green mix (Promega, Madison, WI, USA) and gene specific primers. RNA integrity was tested by PCR amplification of the glyceraldehyde 3-phosphate dehydrogenase (GAPDH) or β-actin as housekeeping gene. PCR products were visualized and identified by image Lab software V5.1 on ChemiDoc XRS+ (BioRad Laboratories, CA, USA) after electrophoresis on 1.5% agarose gel and staining with ethidium bromide. Band intensity was quantified using Image Lab 6.1 software.

### 2.14 Flow-cytometry

Flow-cytometry analysis for cell surface phenotypes was performed after the staining of cells (1×10^5^) with fluorescence labelled antibodies (specific and isotype-matched controls) as per the manufacturer’s recommendation. After incubation for 30 mins at 4°C in dark, labelled cells were washed twice with FACS buffer (0.1% BSA and 0.05% sodium azide in PBS) before being analyzed by flow-cytometry.

Similarly, intracellular molecules were stained with fluorescence-labeled antibodies using Cytofix/Cytoperm reagents per the manufacturer’s instructions (BD Biosciences, SanDiego, CA, USA).

Stained cells were fixed with 1% paraformaldehyde in PBS, and acquisition was performed using a FACSCalibur (Becton Dickinson, NJ, USA). Data was analyzed with Cell Quest (Becton Dickinson, Mountainview, CA, USA) and FlowJo (Tree Star, Ashland, OR, USA) softwares. Suitable negative isotype controls were used to establish background staining profile. Gating strategies are provided in the respective figures.

### 2.15 Rhodamine influx assay

The Rhodamine influx assay was performed with cells (1x10^5^) of EP, LP of cancer cells (here, Jurkat cells) by seeding into 6 well tissue culture treated plates and incubating with 3µM rhodamine 123 (Rho123) in presence/absence of MDR inhibitor cyclosporine (10 µM) for 1 hr at 37°C. The rhodamine fluorescence was determined by FACSCalibur flow-cytometry and analyzed with FlowJo software.

### 2.16 Immunofluorescence microscopy

Biopsy tissue samples of NHL patients were prepared (paraffin-embedded) and 5µm sections were stained as per protocol. Briefly, the sections were washed in xylene for 10 mins followed by dehydration with graded alcohol (90% to 50%) and blocked in 5% BSA for 2 hrs, and then overnight incubated with primary antibodies in dilution range from 1: 200 to 1: 500, followed by incubation with fluorochrome-tagged secondary antibodies. Slides were mounted with Fluoroshield-DAPI (Sigma-Aldrich, MO, USA). Images were acquired using Fluorescence-Microscope (BX-53, Olympus, Japan). Mean fluorescence intensity (MFI) was analyzed using Fiji-ImageJ software (21).

### 2.17 Cytokine quantification by ELISA

TCM and MDSC supernatants were collected and kept frozen at -80°C. Serological levels of IL-6, IL-2, IL-4, IL-1β, IL-10, IL-13, IL-12, TNF-α, IFN-γ, TGF-β and VEGF were quantified by ELISA and optical density was measured at 450 nm using microplate reader Spectramax i3x (Molecular Devices, San Jose, USA). Data was accounted via SoftMax Pro 7.1 software.

### 2.18 Cytosolic and nuclear lysate preparation

Cells from culture were harvested and then centrifuged at 2000 rpm for 10 mins. The cell pellet was re-suspended in ice-cold nuclear extraction buffer and incubated for 1 hr at 4°C. The cells were then centrifuged at 6000 rpm for 5 mins. The supernatant was isolated as the cytosolic fraction. The remaining pellet was dissolved in nuclear extraction buffer and kept in vortex for 30 mins at 4°C. The suspension was centrifuged at 12,000 rpm for 10 mins. The supernatant was collected as the nuclear fraction (22). The separation was validated by western blot analysis of ‘house-keeping marker’ proteins (nuclear HISTONE H1 and cytosolic GAPDH).

### 2.19 Total protein isolation and Western blot

Cell lysates were prepared by incubating cells from different treatment groups with RIPA buffer for 30 mins and centrifuged at 12,000 rpm for 30 mins. Protein concentrations of cell lysates were determined by Bradford assay. The cellular lysate (total protein or nuclear or cytosolic protein at a concentration 30–50 μg) was separated on 12% SDS-PAGE and transferred onto a nitrocellulose membrane using the BioRad Gel Transfer system. Western blotting was performed with the ECL Kit (Advansta, CA, USA). Band intensity was quantified using Image Lab 6.1 software (Bio-Rad, California, USA).

### 2.20 Cytoplasmic extraction for GSTs (Glutathione S Transferases) and GSH (Glutathione)

Approximately 1X10^6^ cells were taken per extract and washed the cells gently with PBS. After that, cells were centrifuged at 1000 rpm for 5 mins. Supernatant was removed and resuspended the pellet in 5 time volumes of Cytoplasmic Extraction (CE) buffer, composed of 10 mM HEPES, 60 mM KCl, 1 mM EDTA, 0.075% (v/v) NP40, 1 mM DTT and 1 mM protease inhibitor, adjusted to pH 7.6. The mixture was incubated on ice for 3 mins and centrifuged at 1500 rpm for 4 mins. The supernatant was considered as cytoplasmic extract.

### 2.21 Measurement of cellular GSH

GSH was measured according to the method of Sedlak and Lindsay (1968) (23). Cells were treated with cytoplasmic extraction buffer for cellular protein analysis and supernatant (5.0 ml) was mixed with 4.0 ml distilled H_2_O and 1.0 ml of 50% trichloroacetic acid (TCA). The tubes were shaken intermittently for l0-15 mins and centrifuged for 15 mins at approximately 3000 g. Supernatant (2ml) was mixed with 4.0 ml of 0.4 M Tris buffer, pH 8.9 and 0.1 ml DTNB was added, followed by shaking. The absorbance was read within 5 mins of the addition of DTNB at 412 nm against a reagent blank with distilled water. Appropriate standards were taken and protein was measured by Bradford assay.

### 2.22 Measurement of cellular GST

GST activity was determined spectrophotometrically according to the method (22–24) using 1-chloro-2, 4-dinitrobenzene (CDNB) as a substrate. Glutathione conjugates formed in the presence of the enzyme were quantified spectrophotometrically and the specific activity was expressed as nmol/min/mg of protein. Cells were treated with cytoplasmic extraction buffer for cellular protein analysis and the supernatant was considered as sample. For GST estimation, 100 µl sample was taken and mixed with 0.3(M) phosphate buffer (1 ml), CDNB (100 µl) and distilled water (1.7 ml). This was kept in 30°C for 5 mins. After that, GSH (100 µl) was added just before measurement of absorbance taking reading at 340 nm at 30 sec interval.

### 2.23 Cytokine neutralization assay

To neutralize the effect of responsible cytokines (IL-6, IL-10, IL-1β, VEGF) present in MDSC culture supernatant, supernatants were treated with anti-IL-6, anti-IL-10, anti-IL-1β, anti-VEGF antibodies (0.5-5 µg) alone or in combination for 4 hrs to neutralize. Then drug resistant cancer cells (EP and LP) were cultured with treated supernatant in 6 well plates at 1x10^5^ cells/well for 48 hrs. The expression of ABC transporter (Pgp, MRP1) was measured by flow-cytometry. To confirm the effect of cytokines, we treated cells with different recombinant human cytokines (concentration added based on respective cytokine quantification level) for 48 hrs at 37˚C, under 5% CO_2_.

### 2.24 *In vitro* generation of wild type (wt^+/+^) and IL-10 deficient (IL-10^-/-^) MDSCs

Bone marrow cells from wt^+/+^ and IL-10 deficient (IL-10^-/-^) mice were harvested, and to obtain bone marrow derived MDSCs, 2.5 x 10^5^ cells were plated on dishes (100 mm) in 10 ml of complete medium, consisting of 10% FBS, 1 mM sodium pyruvate, 10 mM HEPES, 2 mM glutamine and 50 mM β-mercaptoethanol (Sigma-Aldrich, MO, USA) in DMEM base. The medium was supplemented with GM-CSF and IL-6 (40 ng/ml each) to generate MDSCs and maintained at 37°C in 5% CO_2_-humidified atmosphere for 4 days. The cells were washed twice before any experimentation.

### 2.25 Magnetic cell separation for mouse MDSCs

MDSCs were harvested from mouse tumors by magnetic sorting. The cells were subjected to anti-CD11b (biotinylated) antibody and incubated at 4°C. After 30 mins of incubation, cells were mixed with streptavidin DM particle for 30 mins at 4°C and CD11b^+^ fraction was collected. This was followed by incubation of CD11b^+^ cells with anti-Gr1-DM particle tagged antibody for 30 mins at 4°C. CD11b^+^Gr1^+^ fraction was collected. Cell purity of collected positive fraction was checked by flow-cytometry.

### 2.26 siRNA mediated *in vitro* silencing

siRNA for human-STAT1 and human-STAT3 were constructed *in-vitro* using siRNA construction kit (Life Technologies, USA). The primers used in this purpose are mentioned below; those were constructed according to the manufacturer’s protocol. Both the target specific siRNAs and scramble control siRNA were added to *in-vitro* setup to a final concentration of 50nM. Transfection was conducted using lipofectamine-2000 reagent (Invitrogen, USA) to the 2hr serum starved cells. Cells were harvested 48 hrs post-transfection for RT-PCR and co-culture assays.

STAT3 si F: 5’-AAAGAAACTGCCGCAGCTCCACCTGTCTC-3’.
STAT3 si R: 5’-AATGGAGCTGCGGCAGTTTCTCCTGTCTC -3’.
STAT1 si F: 5’-AACCAAAGAAAAGCGACTATACCTGTCTC -3’.
STAT1 si R: 5’-AATATAGTCGCTTTTCTTTGGCCTGTCTC -3’.

### 2.27 Co-immunoprecipitation assay

To study the interaction between Pgp, MRP1 with NFκB1, NFκB2 domain of NF-κB signalling, co-immunoprecipitation was performed. In brief, 50 μg of protein lysates were pre-cleared by centrifugation at 20,000 g for 10 mins, incubated overnight with primary antibodies or control IgG at 4°C, followed by incubation with protein G Sepharose beads for 2 hrs. The immune complexes were boiled with SDS-sample buffer, subjected to electrophoresis and co-immunoprecipitated proteins were analyzed by western blotting, using ECL Kit (Advansta, CA, USA). Band intensity was quantified using Image Lab 6.1 software.

### 2.28 Chromatin immunoprecipitation assay

ChIP assays were conducted following the manufacturer’s protocol (Millipore, Darmstadt, Germany). Briefly, cells (1×10^5^) were seeded in 2% media for 2 hr and then fixed in 1% paraformaldehyde. Cells were washed with PBS containing 1 mM PMSF and lysed in SDS-lysis buffer. DNA was sheared by ultrasonication (Hielscher, NJ) at 30 kHz and 10 cycles of 30 ON/OFF pulse, followed by overnight incubation with primary antibody p50 and p65 (5 μg), which was then captured using anti-goat IgG-coated agarose beads (Sigma-Aldrich, MO, USA). Eluted DNA was extracted using phenol/chloroform and precipitated with ethanol. 35 cycles of PCR were performed using promoter specific primers for NF-κB binding site of human Pgp and MRP1 promoters.

### 2.29 PPI network visualization

We used the online tool STRING http://www.string-db.org (25), to establish interactome maps of expressed genes in our study (STRING v.11.5). The indicated network properties include: nodes: number of proteins in the network; edges: number of interactions; node degree: average number of interactions; clustering coefficient: indicates the tendency of the network to form clusters.

### 2.30 Statistical analysis

All patient samples were randomized prior to group allocations. All reported results were presented as mean ± standard deviation (SD) of data obtained from patient samples (n=61 for NHL and n=10 for healthy individuals). Statistical significance was performed from one-way or two-way analysis of variance for comparative analysis.

For *in-vitro* and *in-vivo* experiments, all reported results were presented as the mean ± SD of data obtained from statistical analysis of either six for *in-vivo* or three to six for *in-vitro* independent experiments. Survivability of mice was assessed from Kaplan-Meier survival analysis. Statistical significance was established from one-way/two-way ANOVA. All statistical analysis and heat maps were generated by GraphPad Prism 8.4.2 software (GraphPad Software, San Diego, CA, USA). The number of *in-vitro* or *in-vivo* experiments used for each analyses and particular statistical test used are indicated in each figure legend. Experimental groups attaining p ≤ 0.05 considered as significant.

## 3 Results

### 3.1 Elevated level of MDSCs is positively correlated with relapse of NHL patients after R-CHOP therapy

Clinical responses to R-CHOP therapy in NHL patients have been differential with longer complete response rates, higher therapy failure and cancer recurrence. Therefore, a better understanding of this differential response of NHL patients on receiving R-CHOP therapy would be a benefit for the betterment of lymphoma management. Approximately 30% to 50% of NHL patients fail to respond to standard R-CHOP therapy depending on disease stage and prognostic index. Among these non-responder NHL patients, 20% suffer from primary refractory disease (progress during or right after R-CHOP therapy), whereas 30% patients suffer relapse or recur (early relapse within 6 months or late relapse after 2 year of completion of treatment) after achieving complete remission (CR).

**Figure 1:**
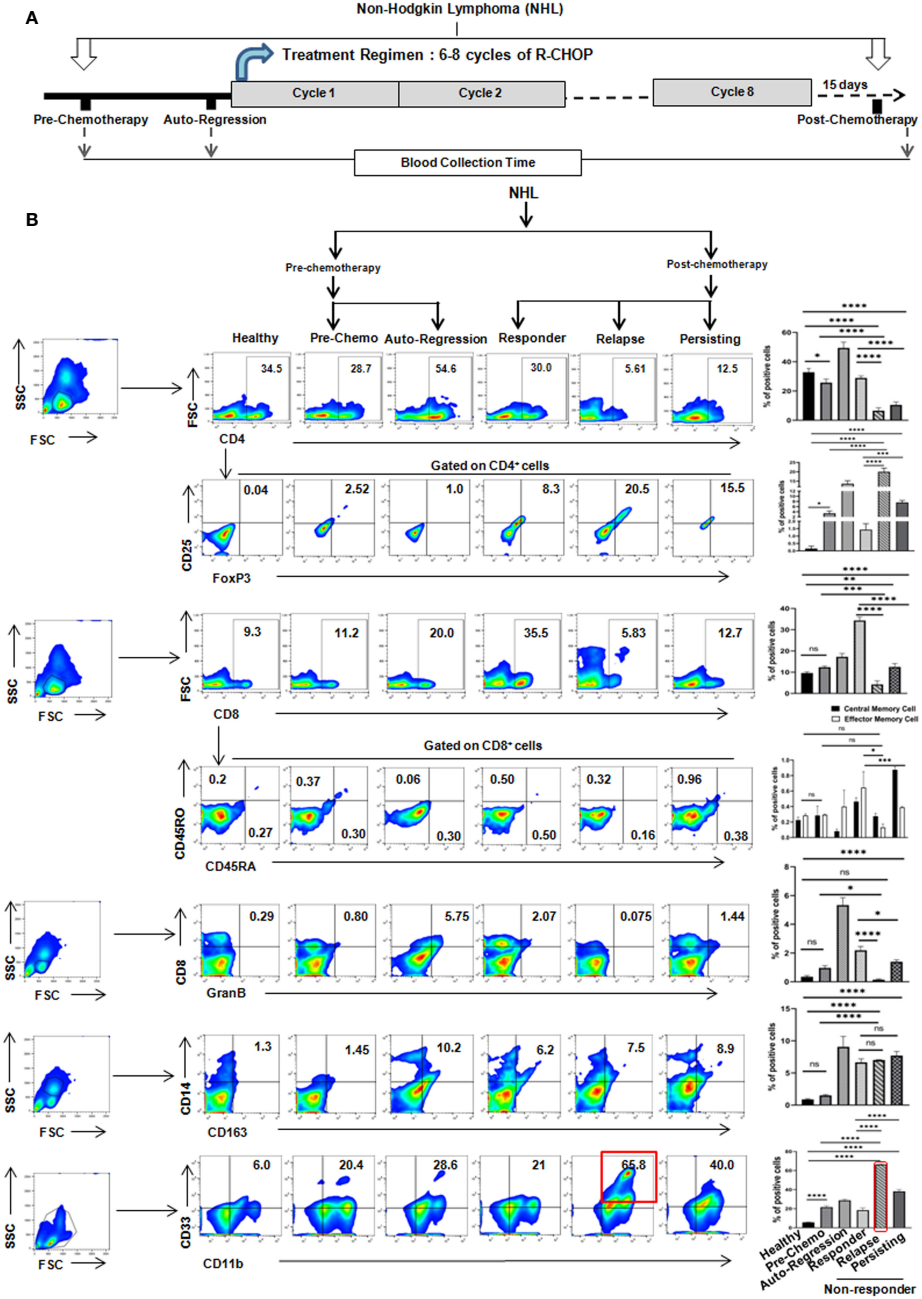
Relapse of NHL patients after R-CHOP therapy correlates positively with frequency of MDSCs: **(A)** Schematic representation of treatment regimen (6-8 cycles of R-CHOP) of NHL patients and blood collection points (pre-chemotherapy, spontaneous regression and post-chemotherapy). **(B)** Representative pseudo color flow-plots showing immunophenotyping of NHL blood samples collected from every group of patients. Bar diagrams represent individual percentage of respective populations with mean ± SD in all groups (n=61 for NHL, where pre-chemo, n=10 and post-chemo, n=51) and healthy individuals, n=10; one-way ANOVA analysis followed by Tukey’s multiple comparison test was done for T cell, GranzB, Treg, M2 macrophages and MDSC populations. Two-way ANOVA analysis followed by Tukey’s multiple comparison test was done for central and effector memory cell population. *p<0.05, **p< 0.01, ***p< 0.001, ****p< 0.0001, ns: not significant are indicated.

So, to investigate the reason behind the differential response following R-CHOP therapy, we have immunoprofiled PBMCs obtained from NHL patients’ blood having both pre-chemotherapy (n=10) and post-chemotherapy (n=51) after 15 days from completion of R-CHOP therapy along with age and sex matched healthy individuals (n=10) (**Figure 1A**). Flow-cytometric analysis of T cells showed a significant decrease in CD4^+^helper, CD8^+^ and CD8^+^GranzB^+^ effector T cell population in relapse/non-responder group than responder patients (**Figure 1B**) No significant alterations were found in case of central memory T cells (CD8^+^CD45RA^+^) and effector memory T cells (CD8^+^CD45RO^+^) among different groups (**Figure 1B**). On the other hand, only modest difference was observed in status of immune suppressor cells, Tregs (CD4^+^CD25^+^FoxP3^+^) and M2 Macrophages (CD14^+^CD163^+^) among non-responder and responder group (**Figure 1B**). Strikingly, a significant elevated frequency (**approx. 60-65%**) of CD11b^+^CD33^+^ MDSCs were found in non-responder patients, particularly in patients with disease-relapse, compared to pre-chemotherapy group **(approx. 20%)** (**Figure 1B**). Notably, percentage of MDSCs increases after R-CHOP therapy and it is found to be lower in blood obtained from healthy individuals and pre-chemotherapy NHL patients than other cohorts of NHL patients. However, a remarkable positive correlation between elevated level of MDSCs and relapse after R-CHOP therapy was found in NHL patients.

### 3.2 Proportion of monocytic(but not granulocytic) MDSCs, low LMR and high MDR markers are positively correlated with recurrence or relapse of NHL patients

**Figure 2:**
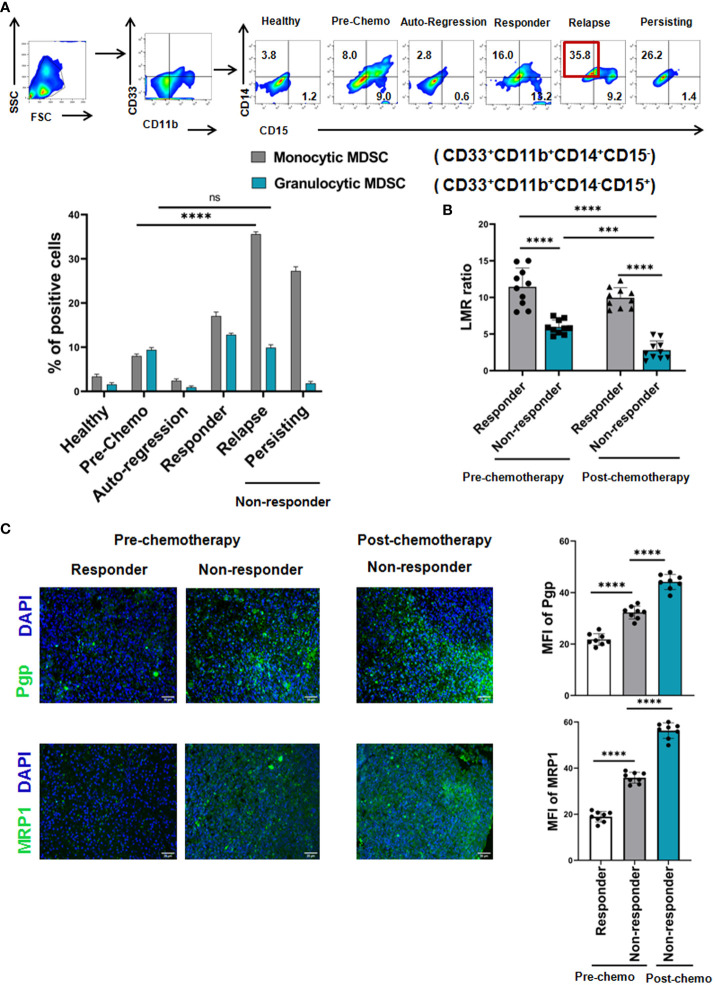
Monocytic, but not Granulocytic, MDSCs induce cancer recurrence of NHL patients after R-CHOP therapy: **(A)** Representative pseudo color flowplots show MDSC sub-typing (M-MDSC: CD33+CD11b+CD14+CD15-, G-MDSC: CD33+CD11b+CD14-CD15+) of NHL patients (pre-, postchemotherapy along with healthy individuals). Blood samples were collected from every group of NHL patients and flow-cytometric staining was done on PBMC. Bar diagrams represent individual percentage of respective populations with mean ± SD in all groups (n= 61 for NHL where prechemo, n=10 and post-chemo, n=51, healthy individuals, n=10). (B) Representative bar diagrams show lymphocyte-monocyte ratio (LMR) of NHL patients (n=40). **(C)** Representative immunofluorescence images of human NHL show the fluorescence intensity of MDR markers measured by Pgp, MRP1 across pre- (n=8) and post-chemotherapy (n=24). One way ANOVA analysis followed by Tukey’s multiple comparison test was performed for **(B, C)** and two way ANOVA for (A) *p < 0.05, **p< 0.01, ***p< 0.001, ****p< 0.0001, ns: not significant are indicated.

Given the strong positive correlation between presences of MDSCs with resistance towards R-CHOP therapy, we have further looked into the importance of MDSC subtypes on disease progression. Two subpopulations, PMN-MDSCs/G-MDSCs - CD33^+^CD11b^+^CD14^-^CD15^+^ and M-MDSCs - CD33^+^CD11b^+^CD14^+^CD15^-^ MDSCs were analyzed in different patient groups along with healthy control by flow-cytometry. A strong elevation of M-MDSCs (approx. 35%) was found in relapse patients compared to pre-chemotherapy patients, where granulocytic MDSCs was only approx. 5-6% in relapse group (**Figure 2A**). Moreover, proportion of M-MDSCs was correlated well with recurrent NHL patients (**Figure 2A**).

In addition to M-MDSC, lymphocyte-monocyte ratio (LMR) is also graded as a critical indicator for clinical outcome of NHL patients. Therefore, LMR at the time of diagnosis, post-chemotherapy and during follow-up period was analysed. Our clinical data shows that in the non-responder cohort, LMR remains low (decrease from 7 to 2 i.e. beginning from pre-chemotherapy till post-chemotherapy) throughout their treatment regimen in comparison to responder cohort (LMR ratio approx. 15). Notably, regardless of response, chemotherapy decreases the LMR in majority of NHL patients. Therefore, low LMR is positively correlated with adverse disease condition, counting as an inexpensive surrogate marker (**Figure 2B**).

Cancer recurrence is one of the global burdens in successful cancer management and 90% of failure in chemotherapy is related to the generation of multi-drug resistance (MDR). So, in line with the clinical data obtained from NHL patients, assessment of NHL cells was conducted in biopsy specimens by fluorescence microscopy which revealed that the increased intensity of MDR marker Pgp and MRP1 in non-responder group in post-chemotherapy phase compared to pre-chemo group (here, post-chemo non-responder groups are the follow up patients of pre-chemo groups), which was strongly associated with the worse treatment outcome (**Figure 2C**). Post-chemotherapy responder groups are exempted from biopsy as they appeared tumor free. Therefore, patients with high expression of MDR markers from the time of diagnosis to completion of chemotherapy might shows more adverse clinical outcome towards R-CHOP-therapy than the patients having low expression of MDR markers in pre-chemotherapy groups.

### 3.3 Monocytic MDSCs promote drug resistance in *in vivo* and *in vitro* setup

**Figure 3:**
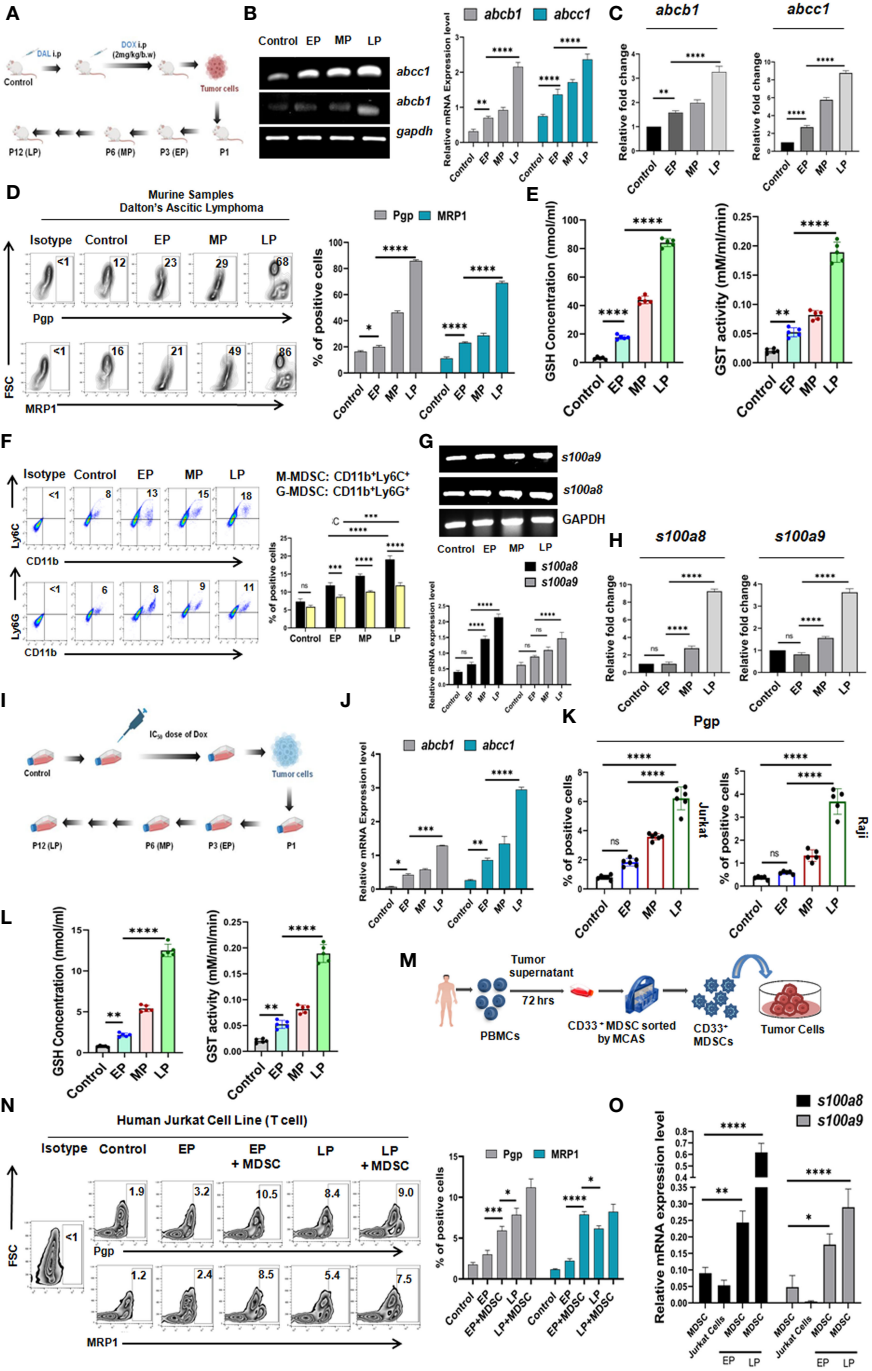
Monocytic MDSC augments drug resistance in *in-vivo* and *in-vitro* drug resistance models: **(A)** Diagrammatic representation of *in-vivo* generation of Dox resistant cancer (Dalton’s Lymphoma or DL) model in Swiss Albino mice. The total *in-vivo* passage was divided into Early (EP), Middle (MP) and Late (LP). **(B)** Gene expression profile of *abcb1*, *abcc1* by RT-PCR and **(C)** Gene expression profile of *abcb1*, *abcc1* by qRT-PCR. Relative fold changes are represented by summary-bars (mean± SD), with statistical significance drawn from One-way ANOVA (n=6). **(D)** Cell surface expression profile of Pgp, MRP1 by flow-cytometry. **(E)** GSH concentration and GST activity by spectrophotometer. **(F)** Percent of M-MDSC (CD11b^+^Ly6C^+^) and G-MDSC (CD11b^+^Ly6G^+^) positive cells by flow-cytometry. **(G)** Gene expression profile of *s100a8* and *s100a9* by RT-PCR. **(H)** Gene expression profile of *s100a8* and *s100a9* by qRT-PCR. **(I)** Diagrammatic representation of *in-vitro* generation of Dox resistant cancer model in T cell (Jurkat) and B cell (Raji) lymphoma. The total *in-vitro* passage was also divided into Early (EP), Middle (MP) and Late (LP). **(J)** Gene expression profile of Pgp, MRP1 by RT-PCR **(K)** Cell surface expression profile of Pgp, MRP1 by flow-cytometry. **(L)** GSH concentration and GST activity by spectrophotometer. **(M)** Generation of MDSCs in *in-vitro* from PBMC of healthy individuals. **(N)** Flow-cytometric zebra-plots (left) and representative bars (right) for MDR expression in EP, MP, LP with and without *in-vitro* generated MDSCs. **(O)** Gene expression profile of *s100a8* and *s100a9* of MDSC in EP, MP, LP by RT-PCR with summary bar-graphs (right). Statistical significance is calculated from one way ANOVA analysis followed by Tukey’s multiple comparison test (n=5) for **(C, E, H)** n=6 for **(K)**, n=5 for **(I)** and two way ANOVA analysis for n=6 for **(B, D, F, G),** n=4 for **(J, N, O)**. *p < 0.05, **p< 0.01, ***p< 0.001, ****p< 0.0001, ns: not significant are indicated.

Given the positive correlation between MDSCs and MDR in NHL patients, their direct interaction was evaluated into *in-vivo* mice model. Initially, mouse Doxorubicin(Dox) resistant Dalton’s Lymphoma (DL) model were developed in Swiss albino mice and maintained upto 12^th^ generation (**Figure 3A**), where increased tumor volume was recorded even after continuous Dox treatment indicating the establishment of drug resistance.

Survivability of mice (EP, MP, LP) was analyzed from the day of inoculation of respective passage cells (description given in materials & methods section) using Kaplan-Meier statistical analysis where mice received longest exposure of Dox (LP) died early (approximately within 15 days after completion of treatment) due to development of drug resistance in comparison to control. The average survival rate of EP was maximum 25 days, in respect to survivability of control mice that ranges 50-55 days in average (**Additional File 3, Supplementary Figure S1**). Next, to investigate the MDR profile, the ascitic fluid of mice from EP, MP, LP passages was harvested and expressions of drug resistance efflux pumps *abcb1* (Pgp), *abcc1* (MRP1) were examined by RT-PCR and flow-cytometry. A gradual elevation of Pgp and MRP1 expression (approx. for Pgp,12%-70% and for MRP1,15%-85%) in protein level along with increment in mRNA data suggests the development of drug resistance in murine *in-vivo* model (**Figures 3B, D**). The upraised expression of abcb1, abcc1 was further confirmed by qRT-PCR (**Figure 3C**). Over expression of Glutathione S-transferases (GSTs) and glutathione (GSH) fosters the detoxification of chemotherapeutic drugs and reduces their cytotoxic effects, thus, promote drug resistance. We observed a surged level of reduced GSH concentration and enzymatic activity of GST which showed direct correlation with generation of drug resistance phenotypes *in-vivo* (**Figure 3E**). Furthermore, in line with the expression of MDSCs in NHL patients, here also, we have analyzed MDSC status obtained from ascitic fluid of EP, MP, LP mice where a significant upsurge of CD11b^+^Gr1^+^ MDSCs with gradual progression of drug resistance was noted (**Figure 3F**). We have also checked the expression level of MDSCs subtypes, both M-MDSC and G-MDSCs in *in-vivo* model, where strikingly a surge of M-MDSCs expression was noted compared to G-MDSCs in the development of drug resistance in mice model (**Figure 3F**). Furthermore, to re-confirm the presence of M-MDSCs, expression of *s100a8* and *s100a9*, two potential markers for this category of M-MDSCs (19) was also found notably higher in mRNA level, confirmed by RT-PCR and qRT-PCR. The expression level of these two markers was gradually higher in MDSC isolated from ascites of LP in comparison to EP (**Figures 3G, H**), validating the association of this type of M-MDSCs with drug resistance.

Given the crucial role in MDSCs in generation of drug resistance, their direct involvement was further validated in *in-vitro* (with Jurkat cells- T cell lymphoma; Raji cells- B cell lymphoma) drug resistance model based on IC_50_ dose of Dox (**Figure 3I**). For this, the cytotoxic activity of Dox was analyzed by the concentration-response curves using MTT cytotoxicity assay. The IC_50_ dose of Jurkat and Raji cell lines was observed to be 2.5 nM and 3.25 nM respectively (**Additional File 4, Supplementary Figure S2**). The gradual elevated expression profile of MDR markers with passages (EP,MP,LP) in both mRNA and protein levels validated the establishment of *in-vitro* generated drug (Dox) resistance model (**Figures 3J–L** **;** **Additional File 5, Supplementary Figure S3A**).

Next, to understand the underlying role of MDSCs in drug resistance and cancer recurrence, CD33^+^ MDSCs were generated from human PBMC *in-vitro* (**Figure 3M**). Then, we directly co-cultured this *in-vitro* generated CD33^+^ MDSCs with cells from EP, LP in a ratio of 1:1 for 48 hrs. Here, addition of CD33^+^ MDSCs with EP cells significantly augments the expression of Pgp and MRP1, which was closely similar to the expression level of MDR markers observed in LP cells (**Figure 3N**, **Additional File 5, Supplementary Figure 3B**).

Furthermore, to confirm the role of responsible subtype of M-MDSC in augmenting early MDR, we have found that the pre and post co-culture sorted MDSCs are actually expressing high *s100a8* and *s100a9* genes compared to cancer cells, suggesting the possible role of M-MDSC in regulating MDR in *in-vitro* set up (**Figure 3O**).

### 3.4 Monocytic MDSCs induced acceleration of drug resistance is contact independent

**Figure 4:**
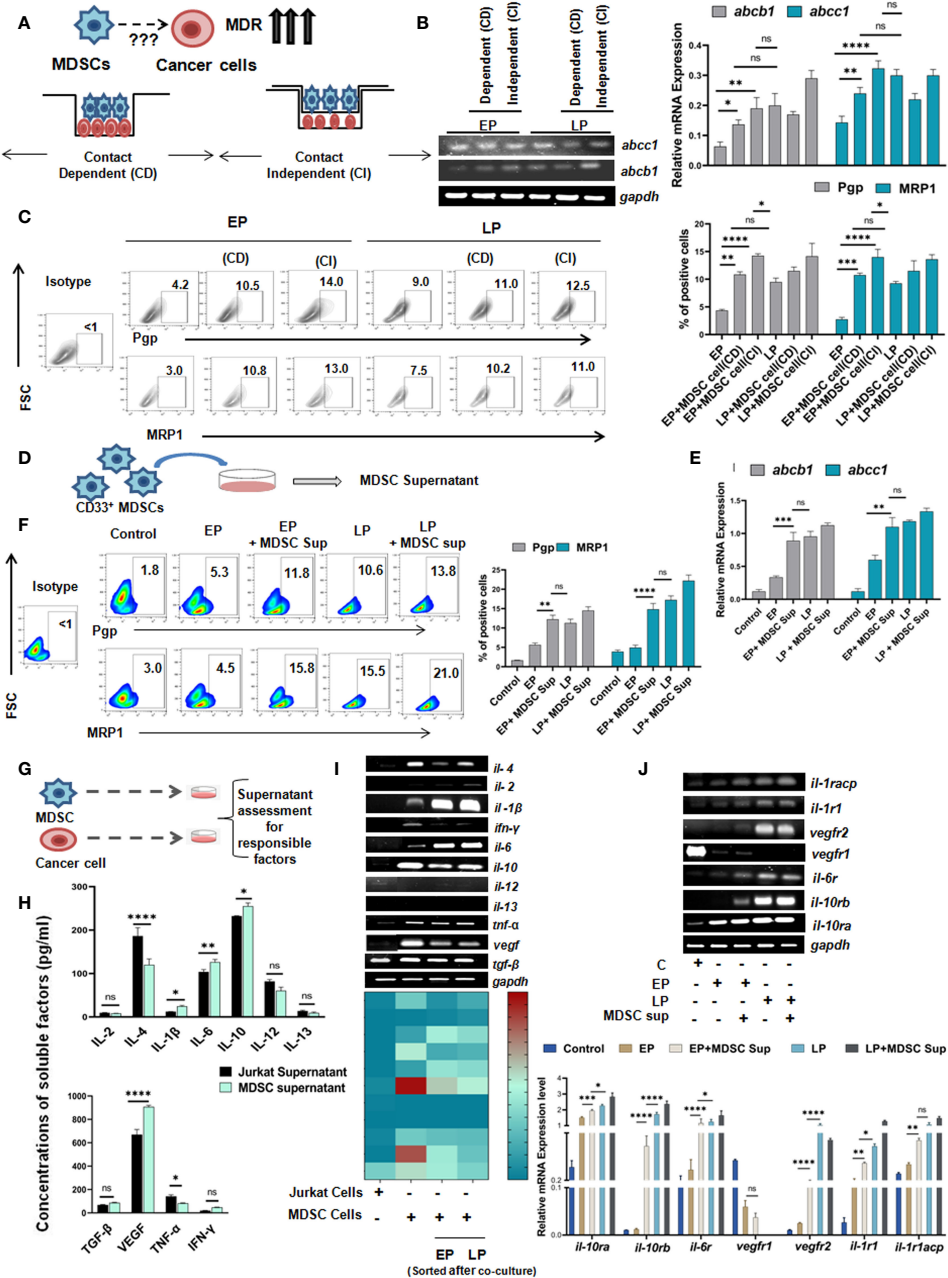
Monocytic MDSCs promote drug resistance through secreted soluble factors: **(A)** Diagrammatic representation of the research question. **(B)** Representation of mRNA expression of MDR-phenotypes *abcb1*, *abcc1* by RT-PCR. Quantified values are represented as relative mRNA expression in bar-diagrams (mean ± SD). **(C)** Representative bar-diagram showing the interaction between drug resistance passages and *in-vitro* generated MDSCs by CD or CI manner. **(D)** Diagrammatic representation showing the generation MDSC supernatant *in-vitro*. **(E)** mRNA expression of MDR-phenotype regulating genes *abcb1*, *abcc1* by RT-PCR. **(F)** Representative pseudo color-plots with or without MDSC supernatant showing the percentage of MDR phenotypes, keeping no drug treatment cancer cells as control. **(G)** Pictorial representation of cancer cell (Jurkat) and MDSC co-culture to assess and quantify *in-vitro* secreted soluble factors by ELISA. **(H)** Assessment of possible anti- and pro-inflammatory cytokines in supernatant of cancer cell and MDSC co-culture by ELISA. Summary-bar-graphs represent the individual percentage of cytokines and growth factors. **(I)** Identification and genetic-profiling of cancer cells (Jurkat), MDSCs and MDSCs sorted from co-culture set up via RT-PCR. Quantified gene expression intensities are represented by heat-maps, with dark color denoting strong expression and light color denoting weak expression. **(J)** mRNA expression of different receptors on cancer cells from similar co-culture set up was analyzed by RT-PCR. Bar diagrams represent individual percentage of respective populations with mean± SD in all groups (n=4), statistical assessment was done by One way ANOVA analysis followed by Tukey’s multiple comparison s(n=4) test for **(H, J)** and for Two way ANOVA analysis followed by Tukey’s multiple comparison (n=4) test for **(B, C, E, F)** *p<0.05, **p< 0.01, ***p< 0.001, ****p< 0.0001, ns: not significant are indicated.

Having the significant influence of MDSCs on early generation of drug resistance, next we aimed to understand whether such effect of MDSCs on cancer cells is contact dependent (CD) (cell-cell contact) or contact independent (CI) (**Figure 4A**). To do so, we have co-cultured *in-vitro* generated MDSCs with cells from passages (EP, LP of Jurkat and Raji cells) in two different experimental settings. In one set up, CD33^+^ MDSCs and cancer cells were cultured in same well to commence receptor-ligand interaction (CD). In another set up, we have conducted assay in Boyden’s chamber or transwell where two cell types are physically separated by 0.4µM membrane so that cellular components can interact via secretory soluble factors (CI) only. Jurkat and Raji cells without presence of MDSCs were taken as control. After 48 hrs of incubation, the flow-cytometric analysis revealed a prominent surge in MDR expression profile of EP, LP cells, those were kept separate by membrane, thus suggesting a contact independent influence of MDSCs. However, similar trend of moderate upregulation of MDR phenotypes was observed in contact dependent manner. Based on the obtained observation, it can be stated that expression of MDR phenotypes following interaction between passages of Jurkat, Raji cells and MDSCs is mediated by secreted soluble factor(s) (**Figures 4B, C**, **Supplementary Figure S3B**).

**Figure 5:**
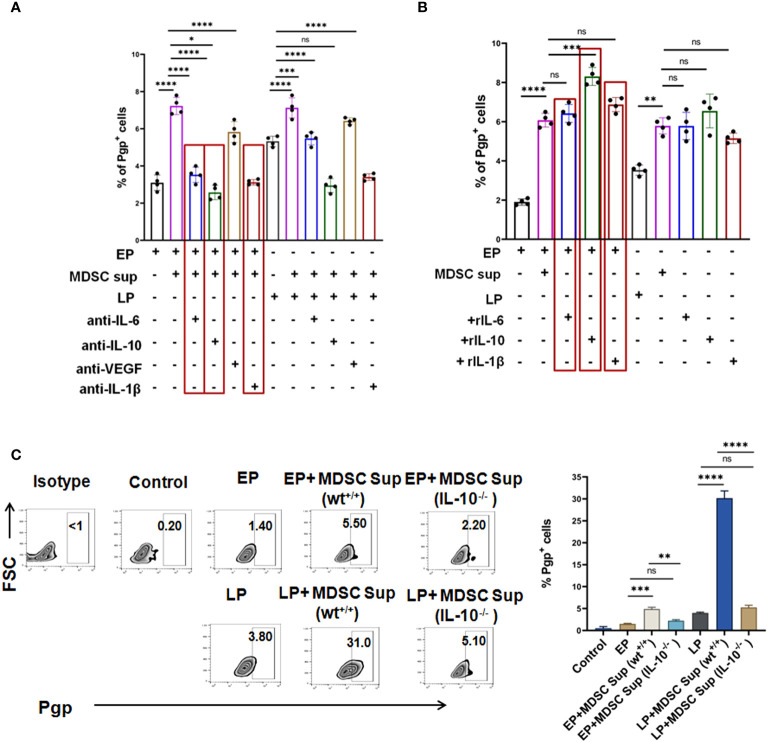
MDSC secreted IL-6, IL-10 and IL-1β co-operatively augment early expression of MDR-phenotype: **(A)** Representative summary-bar showing the upregulated expression of MDR phenotype (Pgp) on EP, LP cancer cells, after addition of MDSC supernatant in presence or absence of cytokines (IL-6, IL-10 and IL-1β). **(B)** Representative summary-bar showing hiked in expression of MDR phenotype (Pgp) of EP, LP cancer cells after exogenous addition of recombinant IL-6, IL-10, IL-1β. **(C)** Representative contour plots showing the effectiveness of MDSC secreted IL-10 on generation of MDR phenotype. EP and LP cancer cells were subsequently treated with bone-marrow derived MDSCs from IL-10^-/-^ mice for 48 hrs, keeping wild type mice as control. Bar diagrams represent individual percentage of respective populations with mean ± SD in all groups (n= 4). One way ANOVA analysis followed by Tukey’s multiple comparison test was performed for **(A–C)** *p<0.05, **p< 0.01, ***p< 0.001, ****p< 0.0001, ns: not significant are indicated.

As MDSC mediated drug resistance is executed via secreted soluble factors, MDSC supernatant was generated by incubation of *in-vitro* generated MDSCs in culture media for 48 hrs (**Figure 4D**) and characterized for further investigation. The dose-response curve depicted that at a particular dose of MDSC supernatant or MDSC sup (150 µl), cancer cells showed highest MDR activity. In line with earlier observations, here also, addition of 150 µl MDSC supernatant significantly increased the expression level of MDR phenotype. Moreover, addition of MDSC supernatant caused early generation of MDR phenotype which was re-confirmed in MDSC treated EP cohort compared to LP (**Figures 4E, F**).

Next, to characterize the MDSC supernatant induced early MDR generation, we have conducted *in-vitro* MDR functional assay using rhodamine 123 of EP, LP cells (Jurkat) with/without MDSC supernatant and MDR inhibitor (cyclosporine). In presence of MDSCs, the accumulated rhodamine was found to be significantly lower in tumor cells than in absence of MDSCs. However, addition of cyclosporine caused increased accumulation of rhodamine in drug-resistant cancer cells than in same cells with no inhibitor. However, the increased expression of rhodamine 123 in cancer cells of cyclosporine-MDSC added group confirms the occurrence of drug resistance through MDSC (**Supplementary Figure S4**).

Next, to ascertain the relevance of particular soluble factor(s) in promoting early expression of drug resistance phenomenon, we examined MDSC supernatant for an array of cytokines and growth factors like IL-2, IL-4, IL-6, IL-10, IL-12, IL-13, IL-1β, VEGF, TNF-α, TGF-β, IFN-γ by ELISA, and keeping cancer cell (Jurkat) supernatant as a control (**Figure 4G**). Here, it was observed that the expression of IL-6, IL-10, IL-1β and VEGF were found to be significantly upregulated in supernatant from MDSC in comparison to cancer cell supernatant (**Figure 4H**). The elevated expression of these soluble factors was further re-validated at mRNA level (**Figure 4I**). Here, in case of *il-6, il-10, il-1β*, the expression of respective cytokines in MDSC isolated from EP, LP co-culture setup were increased gradually from early to late passages. However, *vegf* expression yielded no significant difference. Subsequently, expression of receptors of corresponding cytokines and growth factor on cancer cells (Jurkat) was also analyzed. Here, we have found the prominent expression of *il-6, il-10 and il-1β* receptors on respective cancer cells, without any noticeable change in the expression of *vegf* receptor (**Figure 4J**).

### 3.5 MDSC secreted IL-6, IL-10 and IL-1β promotes MDR phenotypes

Given the predominance of IL-10, IL-6, IL-1β and VEGF in MDSC secreted supernatant and increased expression of corresponding receptors on tumor cells following co-culture with MDSC and MDSC-supernatant, next we tried to validate the role of individual cytokine in induction of MDR. To do so, we have first neutralized the supernatant using neutralizing antibodies for each cytokine and incubated these neutralized supernatant with EP, LP cancer cells for 48 hrs and MDR phenotypes were studied by flow-cytometry. Here, we have observed a significant downregulation of the surge of MDR expression due to individual addition of IL-6, IL-10 and IL-1β neutralized supernatant (**Figure 5A**). The lowest activity of MDR was found on neutralizing MDSC supernatant by anti-IL-10 neutralizing antibody (**Figure 5A**). No significant alteration in expression of MDR activity was found on addition of VEGF neutralized supernatant.

In parallel experiments, EP and LP cancer cells were incubated with exogenously added recombinant cytokines, keeping MDSC supernatant added group as control. In line with our earlier observations, addition of rIL-6, rIL-10 and rIL-1β significantly upregulated MDR expression in EP cells, which was even higher than the expression seen in LP cells (**Figure 5B**). The most prominent expression of MDR phenotype was found on addition of rIL-10 followed by rIL-6 and rIL-1β individually in both EP and LP cells.

Having the dominant role of IL-10 in MDSC induced drug resistance generation, next we have harvested bone-marrow cells from IL-10^-/-^ and wt^+/+^ mice and from them, MDSCs were generated *in-vitro*. CD11b^+^Gr1^+^ MDSCs were then magnetically sorted and MDSC supernatant was generated from both wild type and mutant strains of MDSC cultures. EP and LP cells were incubated with MDSC supernatant generated from IL-10^-/-^ and wt^+/+^ mice and Pgp expression was checked by flow-cytometry. As shown in **Figure 5C**, supernatant from bone marrow derived MDSCs of IL-10^-/-^ mice exposed to cancer cells (EP, LP) revealed a substantial reduction in the expression of Pgp than wild type, validating the role of IL-10 in generation of drug resistant cancer cells.

### 3.6 MDSC induced MDR activity is regulated by STAT3/STAT1/NF-κB axis

**Figure 6:**
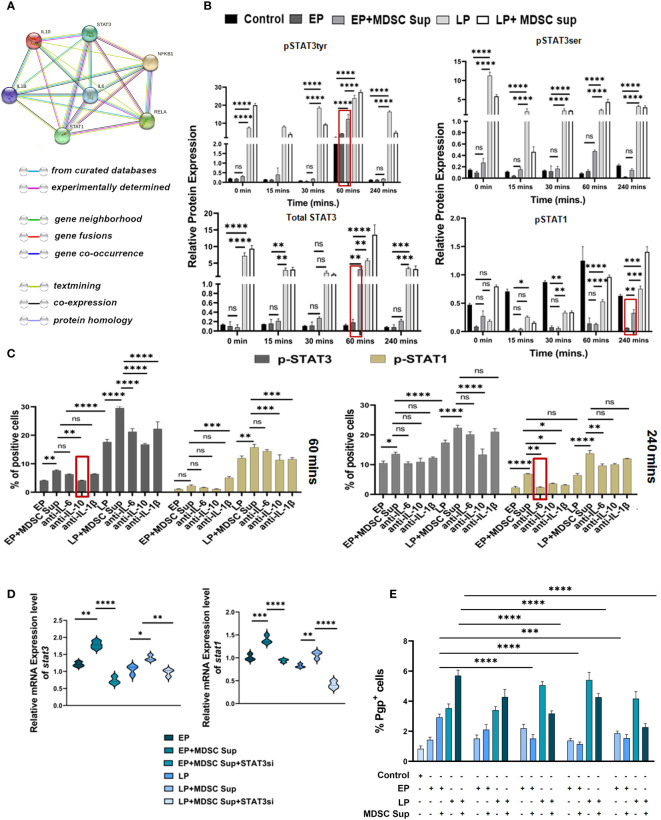
MDSC induced MDR activity is regulated by STAT3/STAT1: **(A)** Representative image showing the interaction of IL-6, IL-10, IL-1β with their transcription factors STAT-1, STAT-3, NF-κB. This interactome image is taken from STRING. **(B)** Representative bar diagram showing the changes of expression level of different transcription factors in time dependent manner. Time-kinetics of western blot data of control (no-treatment), EP, LP cancer cells with/without MDSC supernatant showing the expression level of total STAT3, p-STAT3 (ser), p-STAT3 (tyr), p-STAT1. Representative bar diagram showing individual percentage of respective populations with mean ± SD in all groups (n= 4). **(C)** Illustrative bar diagram showing the MDSC secreted IL-6, IL-10 and IL-1β mediated alteration of transcription factor in time dependent manner. Here, EP and LP cancer cells were co-cultured with MDSC supernatant in presence or absence of neutralized cytokines (IL-6, IL-10 and IL-1β). Bar diagrams represent individual percentage of respective populations with mean ± SD in all groups (n=4). **(D)** Represented bar diagram (mean ± SD) showing mRNA expression of *stat3* and *stat1* gene of cancer cells by RT-PCR. **(E)** Bar diagram displaying % of Pgp population after siRNA-silencing of STAT3 and STAT1 in EP, LP population in presence or absence of MDSC supernatant. Each bar diagram representing mean ± SD of respective cohorts. Statistical significance assessed by One **(D, E)** and Two way **(B, C)** ANOVA analysis followed by Tukey’s multiple comparison (n=4) test done. *p < 0.05, **p< 0.01, ***p < 0.001, ****p< 0.0001, ns: not significant are indicated.

Given the involvement of MDSC secreted cytokines, IL-6, IL-10 and IL-1β in regulation of MDR phenotypes, next we have screened the downstream signaling. First we used STRING database, which predicts protein–protein interaction across a variety of expressed genes. Considering the data obtained from STRING analysis, we have explored cytokine mediated two major signalling pathways namely STAT pathway and NF-κB pathway (**Figure 6A**).

We have treated EP and LP cancer cells with MDSC supernatant, keeping cancer cells without Dox-treatment as control and assessed the status of these transcription factors by western blot analysis in a time-dependent manner (**Figure 6B**). Treatment of EP-cancer cells with MDSC-supernatant significantly upregulated expression of p-STAT3 at 60 mins, which returned to near basal level within 240 mins. The expression of p-STAT3 on EP cancer cells after addition of MDSC supernatant is closely similar to the p-STAT3 expression pattern of drug-resistant LP cells, depicting the role of STAT3 in generation of early drug resistance phenotype (**Figure 6B**). Addition of MDSC-supernatant moderately upregulates expression of pSTAT1 on EP cells at 240 mins in comparison to EP cells and the expression level is at par with LP cells. To further investigate the connection between STAT1, STAT3 and said cytokines in drug resistance, we have treated cells (EP and LP) with cytokine-neutralized (IL-6, IL-10 and IL-1β) MDSC supernatant (in presence or absence of these cytokines), keeping untreated cancer cells as a control. A time course study with IL-10-neutralized MDSCs supernatant on Dox resistant cancer cells, showed downregulated p-STAT3 expression as early as 60 mins post treatment, whereas, IL-6- neutralized supernatant has showed decreased p-STAT1 activation after 240 mins of treatment (**Figure 6C**).

To further validate the role of STAT3 and STAT1 in MDSC-secreted cytokine mediated generation of drug resistance phenomena, we silenced STAT3 and STAT1 siRNA via target specific siRNA on EP & LP cells in *in-vitro* set up. The silenced frequency of STAT3 & STAT1 (approximately 60% & 50% respectively) was confirmed by *in-vitro* flow-cytometric analysis (**Supplementary Figures S5A, B**). The experiment was conducted using silenced and non-silenced EP, LP cells in presence or absence of MDSC supernatant. The data of mRNA of *stat3* & *stat1* silencing groups in presence or absence of MDSC sup confirmed the observation that STAT3 and STAT1 both are crucial constituents in TME promoting drug resistance via MDSC (**Figure 6D**). The flow-cytometric data disclosed that the transient STAT3 and STAT1 knockdown was sufficient to repress the MDR activity of cancer cells exposed to above mentioned condition (**Figure 6E**). The individual silencing of STAT3, STAT1 on cancer cells was much prominent to suppress the MDR activity (**Figure 6E**). Collectively this observation suggests that MDSC augments early generation of drug resistance of cancer cells by IL-6/STAT1 and IL-10/STAT3 axis.

**Figure 7:**
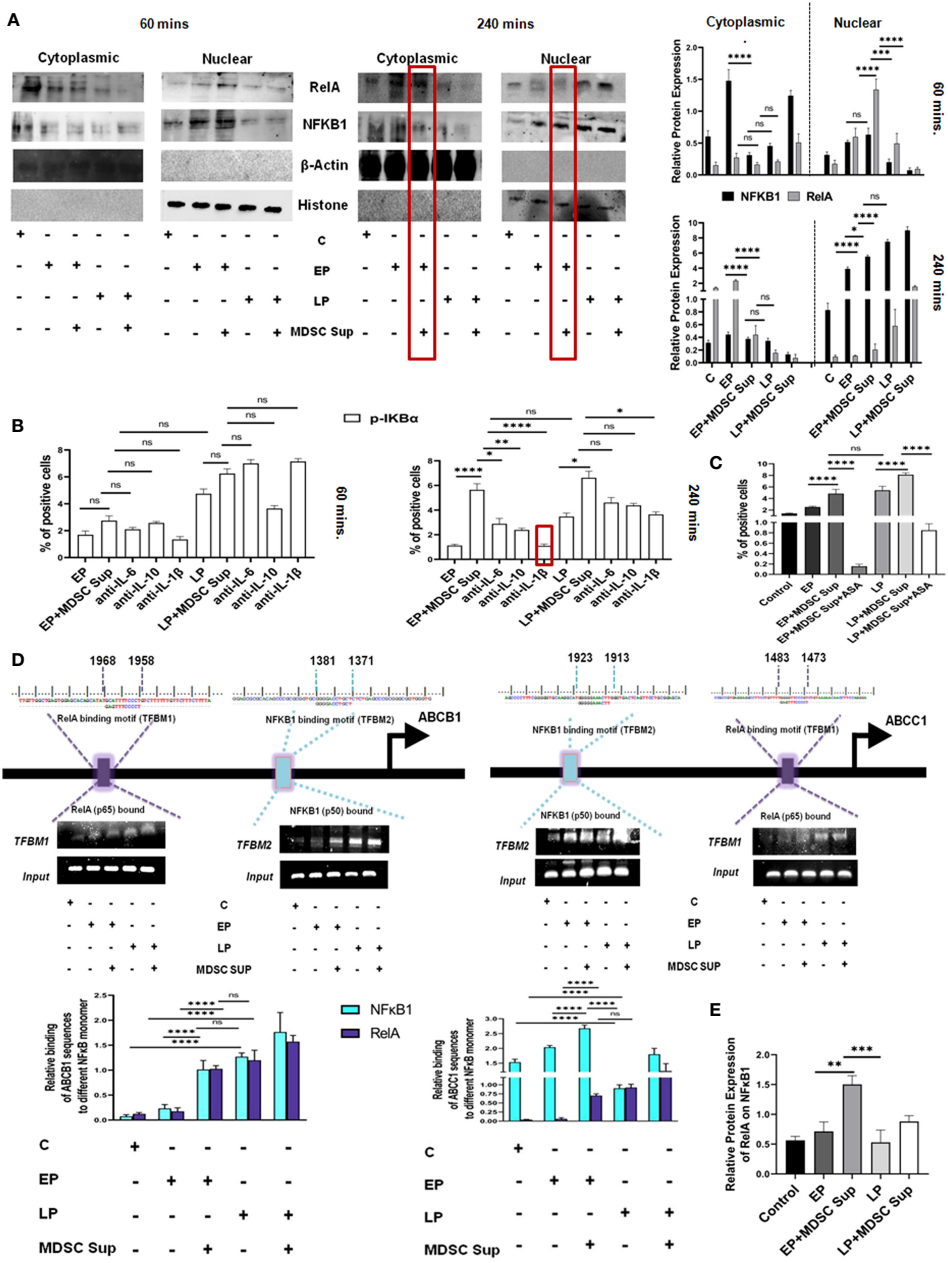
MDSC secreted IL-1β induced MDR activity uses NF-κB axis: **(A)** Representative time-kinetics of western blot data of control (no-treatment), EP, LP cancer cells with/without MDSC supernatant showing the expression of **p50 (NFκB1), p65 (RelA)** in NF-κB signalling pathway. Representative bar diagram showing the changes in expression level of different transcription factors in time dependent manner. Bar diagrams represent individual percentage of respective populations with mean± SD in all groups (n= 3). **(B)** Illustrative bar diagram showing the MDSC secreted IL-6, IL-10 and IL-1β mediated alteration of transcription factor in time dependent manner. Bar diagrams represent individual percentage of respective populations with mean ± SD in all groups (n=4). **(C)** Representative summary-bar showing the % of Pgp of EP and LP cancer cells on treatment with MDSC supernatant in presence or absence of aspirin (ASP). Each bar diagram is representing mean ± SD of respective cohorts. **(D)** Potential NFκB1 and RelA regions within human ABCB1 and ABCC1 gene promoters. Indicated regions are for PCR primer positions used in ChIP assay for NFκB1 and RelA recruitment to the MDR gene. Bars representing mean ± SD of quantified ChIP DNA levels with respect to input. **(E)** Co-IP data shows the status of NFκB1 while pull-down with RelA antibody. The presence of NFκB1 along with RelA confirmed the binding of heterodimer NFκB1-RelA molecule to ABC gene. Bars representing mean± SD of quantified ChIP DNA levels with respect to input. Statistical assessment was done by one-way ANOVA (n=4*)* for **(B, C, E)** and for two way ANOVA (n=4) for **(A, D)** followed by Tukey’s multiple-comparison-test. *p<0.05, **p< 0.01, ***p< 0.001, ****p< 0.0001, ns: not significant are indicated.

Next, to screen another proposed signaling cascade, NF-κB signaling pathway, we have performed an experiment where EP, LP cancer cells were incubated with MDSC supernatant, keeping cancer cells as control and analyzed NF-κB associated transcription factors p50, p65 by western blot analysis. Here, a time-course experiment suggests that MDSC secreted soluble factors actually inducing drug resistance by active participation of NF-κB transcription factor p50 and p65. Furthermore, it was also noted that the cytoplasmic-nuclear transition of transcription factors p50 and p65 in EP, LP passage cells was occurring within 240 mins not in initial hour (60 mins) (**Figure 7A**). Next, to identify the responsible factors activating NF-κB, we have treated cells (EP and LP) with MDSC supernatant in presence/absence of IL-6, IL-10 and IL-1β neutralizing antibodies, keeping untreated cancer cells as a control. This time-course experiment suggests that upon addition of IL-1β neutralized supernatant, significant reduction in p-IKBα activation observed after 240 mins, not in initial hour (**Figure 7B**). So, this finding confirms the role of MDSC secreted IL-1β mediated MDR in cancer cells by activating NF-κB signaling cascade.

To re-confirm the involvement of IL-1β mediated NF-κB signaling in drug resistance, we designed another experimental set-up where EP and LP cancer cells were exposed to aspirin (ASP; 10 mM), an inhibitor of NF-κB activation and MRP1 was evaluated by flow-cytometry. Treatment of EP and LP cells with MDSC-supernatant along with Aspirin failed to upregulate expression of Pgp, validating the role of NF-κB in regulating MDSC induced drug resistance in cancer cells (**Figure 7C**).

To further investigate the transcriptional activation of NF-κB signaling pathway modulating MDR gene, we identified transcription factor binding motif (TFBM) of NFκB1 and RelA on ABC transporter gene (ABCB1, ABCC1) respectively. To directly identify the association of NFκB1 and RelA on MDR gene of cancer cells, we have treated EP and LP cancer cells with MDSC supernatant for a period 240 mins, keeping cancer cells without Dox as a control and then carried out ChIP assay followed by immune-precipitation. NFκB1 (p50) and RelA (p65) were pulled down with anti-RelA and anti-NFκB1 antibodies respectively using ChIP assay and their binding was confirmed by Western Blot analysis (**Figure 7D**). As nuclear-translocation of p50/p65 heterodimer is essential for NF-κB activation, next we checked presence of respective protein partner on MDR gene in mRNA level by RT-PCR. The result confirmed the binding of p50-p65 heterodimer to MDR gene, required from promoting MDR phenotype. Furthermore, this ChIP assay of NFκB1 and RelA in ABCB1 & ABCC1 gene followed by Co-IP and RT-PCR, suggested that the presence of MDSC secreted soluble factor IL-1β in MDSC that actually activating the NF-κB signaling pathway, activating the MDR phenotype, thus augmenting the drug resistance phenotypes (**Figures 7D, E**).

Furthermore to validate the involvement of Stat1/Stat3/NF-κB signaling axis in generation of MDSC-induced drug resistance, we have conducted Rhodamine influx assay with blocker of all three transcription factors. STAT1 and STAT3 were blocked using specific siRNA, whereas aspirin was used to block NF-κB to study the synergistic effect of these pathways. STAT1/STAT3 silenced Jurkat cells were cultured with MDSC supernatant in presence of NF-κB blocker for 48 hrs. The flow cytometry analysis following 1 hr Rhodamine incubation suggested that accumulation of rhodamine was found to be highest with the silencing of STAT1 followed by STAT3 and NF-κB in comparison to MDSC-supernatant treated cells. However, simultaneous blocking of STAT1, STAT3 along with NF-κB shows less accumulation of Rhodamine. Therefore our cumulative results suggest MDSC secreted IL-6, IL-10, IL-1β mediated MDR phenomenon are regulated by STAT1, STAT3, NF-κB individually, not in a combinatorial or synergistic manner (**Supplementary Figures S6A, B**).

## 4 Discussion

In the present study, we assessed 61 NHL patients (fifty one NHL post-chemotherapy patients, along with ten patients with pre-chemotherapy). Among post-therapy patients, twenty four developed R-CHOP therapy resistance within 4-5 months after completion of therapy. Previous studies suggested approximately 60-65% NHL patients including DLBCL can be cured with standard front-line therapy, R-CHOP, while, remaining 30-40% of patients exhibit primary refractory disease or relapse following an initial response to therapy and have shown very poor prognosis with a median overall survival (OS) of less than a year (26). In the second line setting, though autologous stem cell transplantation (ASCT) represents the best available treatment option, majority of the patient found ineligible and such scenario pointing a significant need for deep understanding of NHL from various perspectives for the development of alternative strategies. While numerous studies have explored the genetic variations of NHL at the time of diagnosis, only fewer focused on the identification of immunophenotyping based cellular marker(s) and their link with R-CHOP-therapy resistance.

Immunophenotypic assessment of treatment-refractory/non-responder NHL patients, along with pre-chemotherapy and healthy individuals, have revealed that circulating MDSCs were found to be high in blood of non-responder patients (60-65% MDSC positive cells) compared to responder cohorts. Interestingly, such MDSCs are monocytic (CD33^+^CD11b^+^CD14^+^CD15^-^) in nature (M-MDSC). With enormous pro-tumor abilities, M-MDSCs were found to be elevated in several cancer patients and directly correlated to angiogenesis, chemo-résistance, metastasis and disease advancement (27). Although tumor promoting effects of MDSCs is explored in various forms of cancer (28), impact of chemotherapy induced MDSCs on chemo-resistant cancer cells or generation of chemo-resistance is not studied yet. Moreover, observation from our study reported the high prevalence of M-MDSCs in *de novo* as well as non-responder patient cohort and its strong positive correlation with MDR markers, Pgp and MRP1; its possible contribution is investigated in disease recurrence among NHL patients.

Here, though we haven’t directly assessed how M-MDSC accumulation is more profound but it could be envisaged that altered hematopoiesis might results in a considerable expansion and accumulation of immature myeloid cells (IMCs) in the tumor microenvironment (TME), in secondary lymphoid organs and tissues, driven by tumor-derived growth factors and other soluble factors via STAT1, STAT3, STAT6, COX2 or the NF-κB pathway (29). Tumor-derived GM-CSF, IL-6, IL-1β, TGF-β, IL-13, IFN-γ, CXCL5 have also been found to regulate MDSC accumulation and the polarization of tumor-infiltrating granulocytic MDSC (G-MDSC) toward an immunosuppressive phenotype (30, 31). In contrast, tumor-derived soluble factors M-CSF, IL-6, IL-1β, IL-4, IL-10, CCL2 initiate the immunosuppressive pathways that commit immature myeloid cells to become MDSCs and further promote the differentiation towards M-MDSC (30, 32). Furthermore, we have conducted a pilot study on NHL patients of both responder and non-responder cohorts to study their cytokine profile. This study disclosed the observation that the non-responder NHL patients have a significant upregulation in IL-4, IL-6, IL-10, IL-1β, VEGF, M-CSF expression level with minimum changes in IL-13 and IFN-γ levels. This observation directs towards the accumulation and differentiation of M-MDSC from immature myeloid cells in non-responder cohorts over responder NHL patients. However, further detailed studies are utmost required to decipher the exact reason of M-MDSC over G-MDSC.

To validate the crucial involvement of MDSCs in promoting drug resistance and its mechanism of action, we chose to generate Doxorubicin or Dox resistant tumor models both *in-vivo* with Dalton’s lymphoma (DL) and *in-vitro* with Jurkat-T cell and Raji-B cell lymphoma respectively. Dox treatment for 12 generations in lymphoma bearing mice demonstrated a surge of MDSCs particularly M-MDSCs (CD11b^+^Ly6C^hi^Ly6G^lo^) with increasing drug resistance phenotypes.

To further confirm the direct modulatory effect of M-MDSCs on the promotion of drug resistance, we co-cultured tumor-educated MDSCs along with lymphoma cells (EP, LP of cancer cell line) having pre-exposure of Dox for 48 hrs. *In-vitro* Dox treatment required 12 passages to generate fully drug-resistant cancer cells, termed late passage (LP) or Dox-res passage. Interestingly, addition of CD33^+^ MDSCs in both Jurkat and Raji cell culture accelerated the generation of drug-resistance, as evidenced by significant upregulation of P-gp, MRP expression just only after 3 cycles of Dox-treatment and designated as early passage (EP) cancer cells. This observation clearly pointed out the importance of MDSCs in generation of drug-resistance for the first time to our knowledge. Notably, we observed differential expression pattern of MDR gene in Jurkat cells in *in-vitro* and DL cells in *in-vivo* and consequently, effect of MDSC found to be more higher in case of DL compared to Jurkat cells, which is reported to have low expression of MDR markers in case of Jurkat cells (33). Previous reports suggest the crosstalk between different signaling pathways that can promote Dox resistance via induction of proliferation, suppression of apoptosis, cell cycle progression and altered drug metabolism (34). Growing evidence suggests the important roles of tumor microenvironment including its cellular as well as soluble factors in induction of drug resistance. As MDSCs represent a major constituent of TME, it shows multifaceted abilities in support of tumor progression. In the present work, we are describing its novel role in induction of early drug resistance to cancer cells. In line with earlier reports, accumulation of immunosuppressive M-MDSCs in tumor passage was further supported by the enhanced expression of S100A8 and S100A9, two calcium binding S100 proteins, associated specifically with M-MDSC (35). The secretion of S100A8 and S100A9 by M-MDSCs might provide positive autocrine feedback loop that ensures the maintenance of functionally suppressive MDSCs within an inflammatory tumor environment (36).

At the phase of immune escape, immune system facilitate survival, therapy resistance, selection of resistant cancer variants, and many more by accumulating immune-suppressor cells including regulatory T cells and MDSCs or by releasing tumor promoting cytokines and growth factors (37). In search of the underlying mechanisms, our results suggest that MDSCs instigated generation of early drug resistance in cancer cells via MDSC-secreted soluble factors that act in contact independent manner. Complementing this observation, we found higher expression of IL-10, IL-6, IL-1β in both mRNA and protein levels of MDSCs actually promoting early drug resistance. Earlier reports found M-MDSC and PMN-MDSC utilize different suppressive mechanisms in which M-MDSC suppress anti-tumor immunity mainly through production of NO and tumor-promoting cytokines (38, 39), whereas, PMN-MDSC inhibits anti-tumor immunity through antigen-specific manner (40–42). According to other studies, TME-derived soluble factors involved in mediating drug resistance are VEGF, bFGF, SDF1, IL-6, IL-3, GMCSF, TNF, NO and many others (43, 44). Though, tumor and tumor associated stromal cells secrete all these cytokines in a great extent, we found MDSCs are the major contributors in releasing IL-10, IL-6 and IL-1β over tumor cells itself. Accordingly, supernatant secreted from MDSCs induces a surge of receptor expression for IL-6, IL-10 and IL-1β in cancer cells. Moreover, both exogenous addition as well as neutralization of respective cytokines alone clearly established the role of these three cytokines in MDSC-induced early generation of MDR phenotype in cancer cells. In many cancers, elevated level of serum IL-6 concentration largely correlates with tumor burden, chemo-resistance and poor clinical outcome. An immunoregulatory cytokine IL-10 plays pleiotropic role in attributing both anti-tumor immunity and maintaining immune-suppressive condition (42). High level of IL-10 is also directly attributes to poor clinical outcome of cancer patients (43). In addition, IL-1β contributes to tumor initiation and progression mainly by inducing chronic inflammation, promoting angiogenesis, as well as navigating MDSCs expansion and migration (44). An elevated frequency of IL-1β was positively correlated with the level of M-MDSCs in the peripheral blood of advanced melanoma patients (45).

In search of the downstream pathway involved, we found that MDSC induced drug resistance is occurring via STAT3/STAT1/NF-κB pathway through IL-10/IL-6/IL-1β axis. STAT3 is reported to be a master regulator of cancer hallmarks and it is wildly associated with cell proliferation, resistance to apoptosis, metastasis, immune evasion, tumor angiogenesis, epithelial mesenchymal transition (EMT), chemo-resistance, response to DNA damage, and the Warburg effect (46, 47). There is growing evidence revealed a strong positive correlation between STAT3 activity and immunosuppressive activity of MDSCs. In contrast to STAT3, the reverse effect was found with STAT1 (48). A more recent study stated that the tumor growth and metastasis of head and neck squamous cell carcinoma were much faster in Stat1^–^/^–^ mice than in Stat1^+^/^+^ mice (48). Surprisingly, this observation is in contrast with our present study where we saw that MDSCs secreted cytokines promote STAT1 phosphorylation. Though, in line with our observation, Lara et al. found a crucial role for STAT1 as an oncogenic gene in many cancers including breast and ovarian cancer (48, 49). We have observed that the activation of STAT3 was brought down on neutralization of IL-10 in 60 min, whereas, STAT1 activation was moderately decreased on IL-6 neutralization on 240 min. Silencing of STAT3/STAT1 resulted reduced MDR frequency in cancer cells, suggesting the involvement of both STAT signaling in MDSC-induced generation of chemo-resistance. Apart from STAT signaling, NF-κB signaling are actively involved, as, IL-1β neutralization in 240 min caused reduced expression of p-IKB. Constitutive expression of p-IKB and cytoplasmic-nuclear translocation of p50-p65 suggested that MDSC is inducing drug resistance phenotype via NF-κB pathways as well. According to the binding of NFκB1 and RelA to ABCB1 (Pgp) and ABCC1 (MRP1) promoters, our observation was solidified that NF-κB axis is equally serving a major role to accelerate MDR status along with STAT3/STAT1 axis. Such involvement of NF-κB was again supported its clinical importance in lymphoid malignancies (50). Furthermore, we have re-validated the involvement of these MDR regulating transcription factors by analyzing MDR functional assay (Rhodamine 123 accumulation assay) where we have noted the significant reduction of MDR activity in presence of individual *stat3, stat1* silenced genes and NF-κB blocker in drug resistant cancer cells.

**Figure 8:**
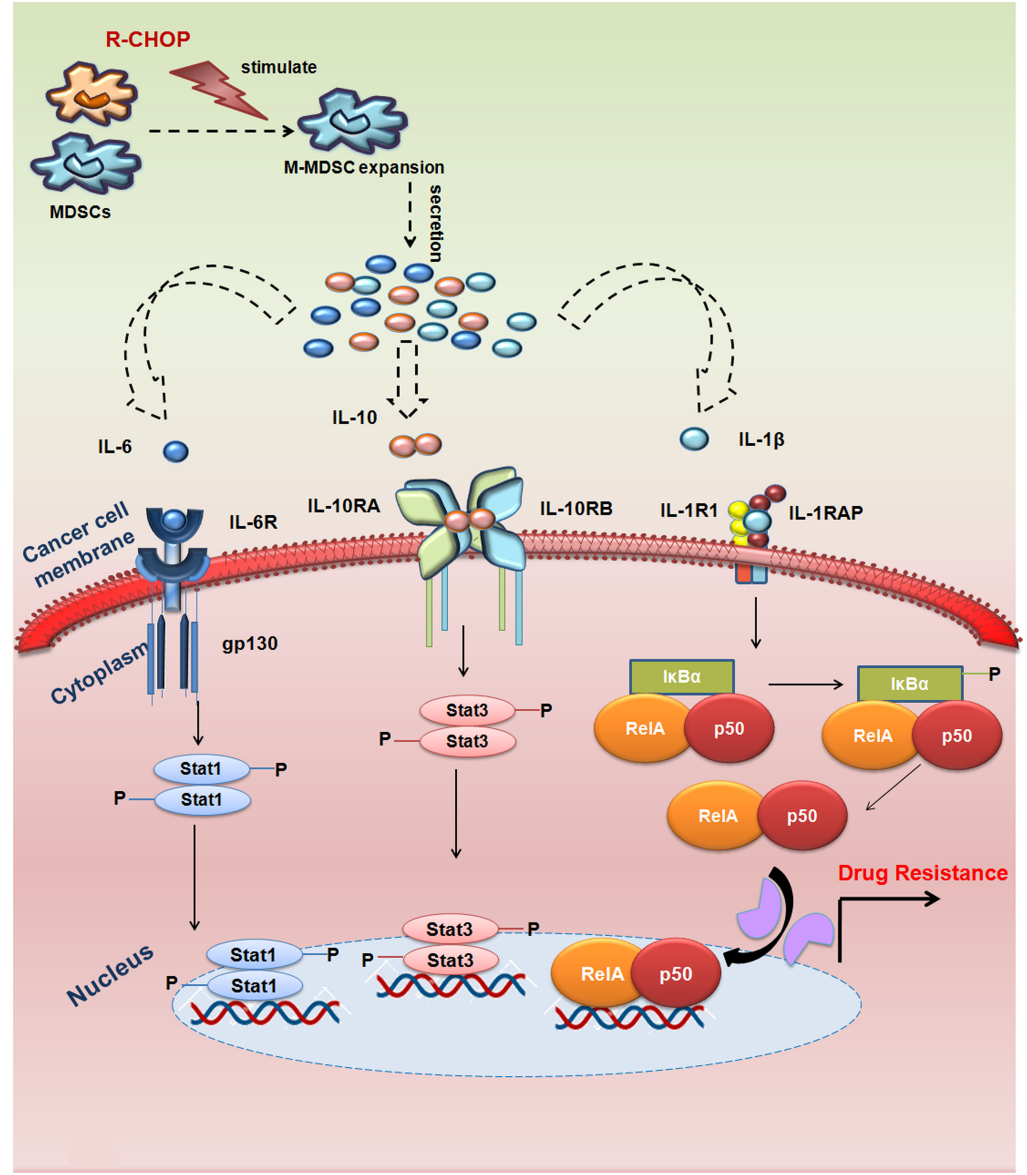
MDSCs induced MDR in NHL relapse patients by modulating IL-6/IL-10/IL-1β axis: R-CHOP treatment in NHL patients leads to expansion of M-MDSC in tumor microenvironment releasing cytokines IL-6, IL-10, IL-1β from M-MDSC cells. These cytokines (IL-6, IL-10, IL-1β), thereby, targeting its respective receptors on the surface of drug resistant cancer cells to activate MDR gene via STAT1/STAT3/NF-κB signalling. This signalling cascade promotes early activation of MDR gene, thereby augmenting drug resistance and cancer recurrence in NHL patients.

Conglomeration of all our observations has suggested that MDSC secreted IL-6/IL-10/IL-1β are the supreme players regulating the drug resistance of cancer cells via STAT1/STAT3/NF-κB pathway (**Figure 8**). Altogether, this study provides a mechanistic view on how M-MDSC promotes early drug resistance in NHL patients even after completing R-CHOP treatment regimen. Abnormal accumulation of M-MDSCs in cancer might help to identify the potential patient group for recurrence. The frequency of monocytic MDSCs can be used as a prognostic biomarker to assess the relapse free survival in NHL patients. Future personalized target-based therapy can also be constructed based on higher presence of MDSCs particularly M-MDSCs in NHL patients. Our study also prospects that targeting STAT3/STAT1/NF-κB axis by neutralizing IL-10/IL-6/IL-1β within MDSCs could also serve as a better therapeutic approach. In future, it would have a significant input in cancer biology to investigate how MDSCs can be modulated functionally to regain immune surveillance in relapse cancer patient cohorts.

## 5 Conclusions

**Figure 9:**
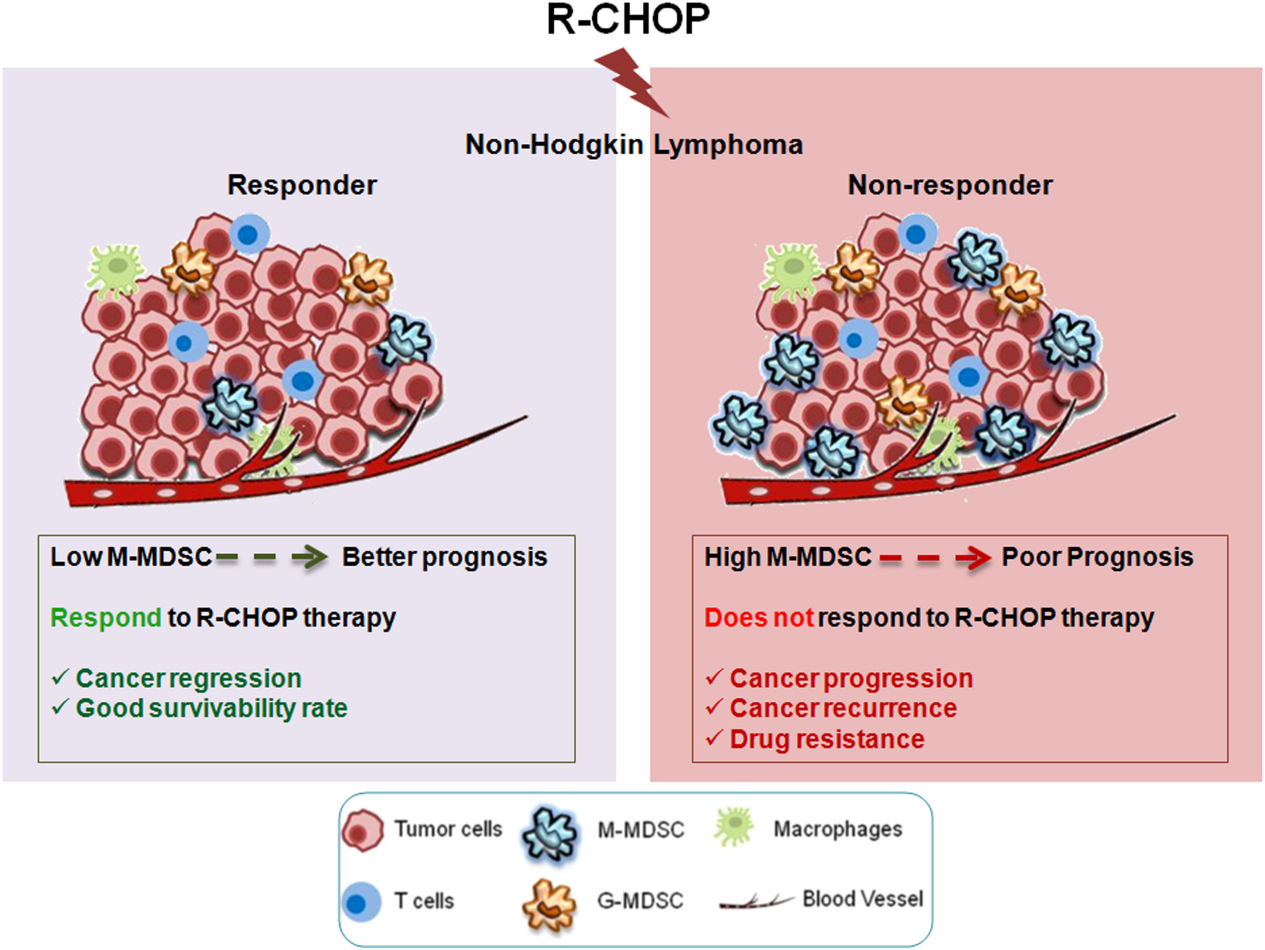
Graphical Abstract: Graphical abstract describing differential response to R-CHOP therapy among NHL patients. Non-responder patients with high M-MDSCs are prone to therapy failure, due to development of drug resistance. Therefore, M-MDSC can be a predictive marker to assess the disease status after R-CHOP therapy. Targeting M-MDSCs can be used as a prognostic biomarker to assess the relapse free survival in NHL patients.

In summary, our study revealed for the first time the dominant role of MDSCs, more specifically M-MDSC in generation of drug resistance in NHL patients undergoing R-CHOP therapy. Altogether, this study highlights a systematic view on how M-MDSC promotes early drug resistance in NHL patients even after completing R-CHOP treatment regimen. Our observations have suggested that MDSC secreted IL-10/IL-6/IL-1β are the critical players modulating the drug resistance of cancer cells via STAT3/STAT1/NF-κB pathway (**Figure 8**). The important observation of this study was the assessment of the frequency of M-MDSCs in non-responder relapse group compared to responder and their direct correlation with the drug resistance status. Furthermore, frequency of M-MDSC can be used as a negative prognostic marker to select NHL patients for R-CHOP (**Figure 9**). In future prospects, targeting M-MDSCs can be a promising approach for Non-Hodgkin lymphoma relapsed patients, which may further refine the potency of current standard R-CHOP therapy.

## Data availability statement

The original contributions presented in the study are included in the article/**Supplementary Material**. Further inquiries can be directed to the corresponding author.

## Ethics statement

The study was approved by the Institutional Human Ethical Committee (IEC Ref: CNCI-IEC-RB-2020-3) to obtain non- Hodgkin lymphoma patient’s blood and biopsy samples. The details are given in methods section. The experimental animal protocol was approved by Institutional Animal Care and Ethics Committee, CNCI, Kolkata (Approval No: IAEC-1774/RB-16/2017/3).

## Author contributions

SDh: Data curation, Formal Analysis, Funding acquisition, Methodology, Software, Validation, Visualization, Writing – original draft. MC: Methodology, Writing – review & editing. NG: Methodology, Writing – review & editing. AkS: Methodology, Writing – review & editing. SDa: Methodology, Writing – review & editing. SBe: Methodology, Writing – review & editing. AnS: Methodology, Writing – review & editing. KR:. JD: Methodology, Writing – review & editing. ABh: Methodology, Writing – review & editing. SG: Methodology, Writing – review & editing. MS: Methodology, Writing – review & editing. SH: Resources, Writing – review & editing. SBa: Methodology, Writing – review & editing. CP:. BS: Resources, Writing – review & editing. KM: Resources, Writing – review & editing. RB: Funding acquisition, Investigation, Project administration, Resources, Supervision, Visualization, Writing – review & editing. ABo: Conceptualization, Funding acquisition, Project administration, Resources, Supervision, Visualization, Writing – review & editing.
