# Supplementary material for: High monocytic MDSC signature predicts multi-drug resistance and cancer relapse in non-Hodgkin lymphoma patients treated with R-CHOP
Source: Front Immunol. 2024 Jan 18;14:1303959. doi: 10.3389/fimmu.2023.1303959 (PMC10831358; doi:10.3389/fimmu.2023.1303959)
Supplement: Supplementary file 1 [file DataSheet_1.pdf]

**Supplementary Figure 1:**

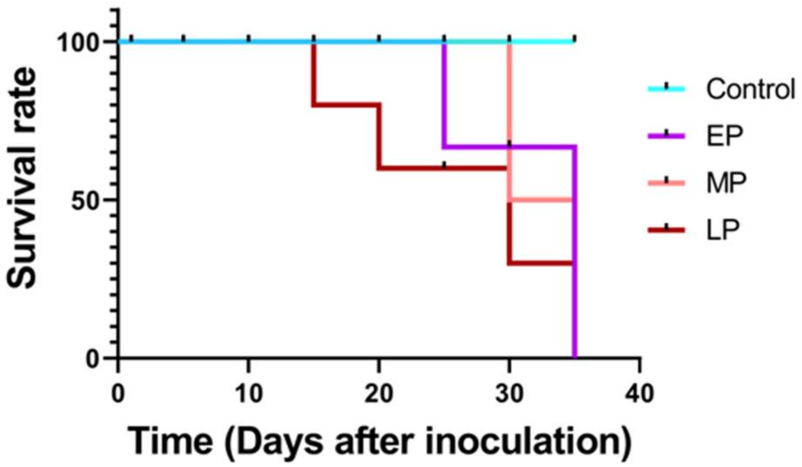


Fig. S1 : Dhar *et al.*,

**Supplementary Figure 1**: Representative graph showing % survivability of drug resistant passages (EP, MP & LP) by Kaplan-Meier test (n=6). *In-vivo* Dalton's Lymphoma (DL) resistance (resistant to Dox) model was developed and maintained upto 12$^{th}$ generation. Total 12$^{th}$ passages were divided as time points by early (EP/P3), middle (MP/P6) and late (LP/P12) passages, illustrated already in **Fig. 3a**. DL cells were inoculated in mice and respective drug (Dox) was successively given for 10 days after 24hrs of tumor inoculation. On 11$^{th}$ day, DL cells were harvested and inoculated to another set of mice naming as P2 (considering inoculation of the previously generated cells from P1). Mice without drug treatment considered as control (C). So, in-short EP mice were inoculated with the previously generated P2 passage cells. Similarly, MP was treated with P5 cells and LP with P11. So, the survival graph analysis of EP, MP, LP were calculated from the day of inoculation previously generated drug resistance cells.

**Supplementary Figure 2:**

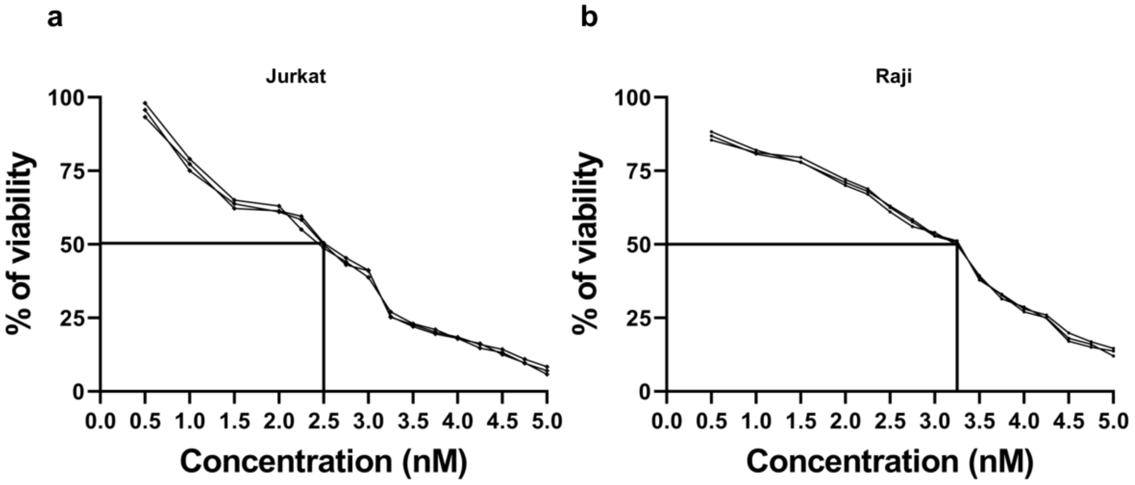


Fig. S2 : Dhar *et al*.,

**Supplementary Figure 2**: Representative cytotoxicity graph of Dox on **(a)** Jurkat **(b)** Raji cell lines. Concentration dependent cytotoxicity of DOX assessed by MTT assay (n=3).

## Supplementary Figure 3:

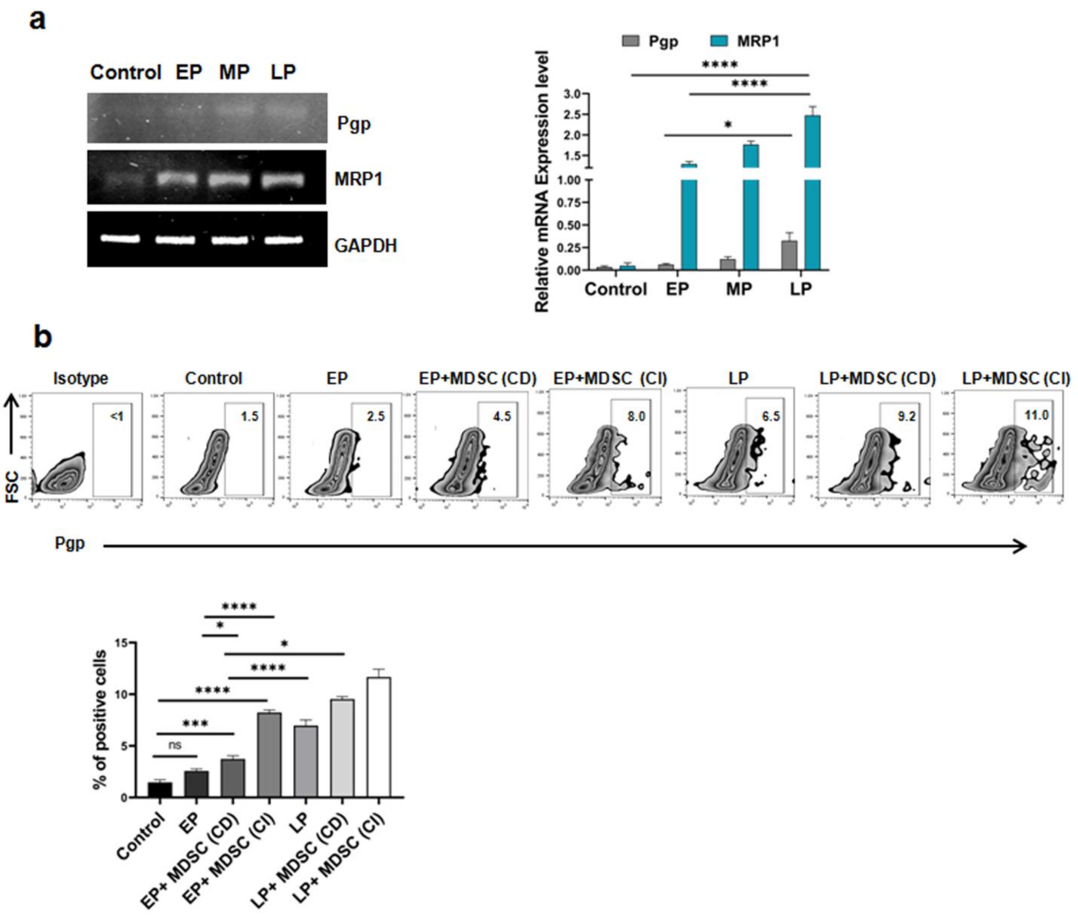


**Fig. S3 : Dhar *et al.*,**

**Supplementary Figure 3**: **(a)** Representation showing mRNA expression of MDR-phenotypes (Pgp and MRP1) of Raji cells by RT-PCR, keeping GAPDH as loading control. Quantified values are represented as relative mRNA expression in bar-diagrams (mean±SD), statistical significance was assessed by One way ANOVA analysis followed by Tukey's multiple comparison (n=4) test. **(b)** Representative zebra plots showing the interaction between different groups of cancer cells in various drug resistance passages (EP, LP) and *in-vitro* generated MDSCs by either contact dependent (CD) or contact dependent (CI) method. Bar diagrams represent individual percentage of respective populations with mean± SD in all groups (n= 4). One way ANOVA analysis followed by Tukey's multiple comparison test done. **p<0.05, **p< 0.01, ***p< 0.001, ****p< 0.0001*, ns: not significant are indicated.

**Supplementary Figure 4:**

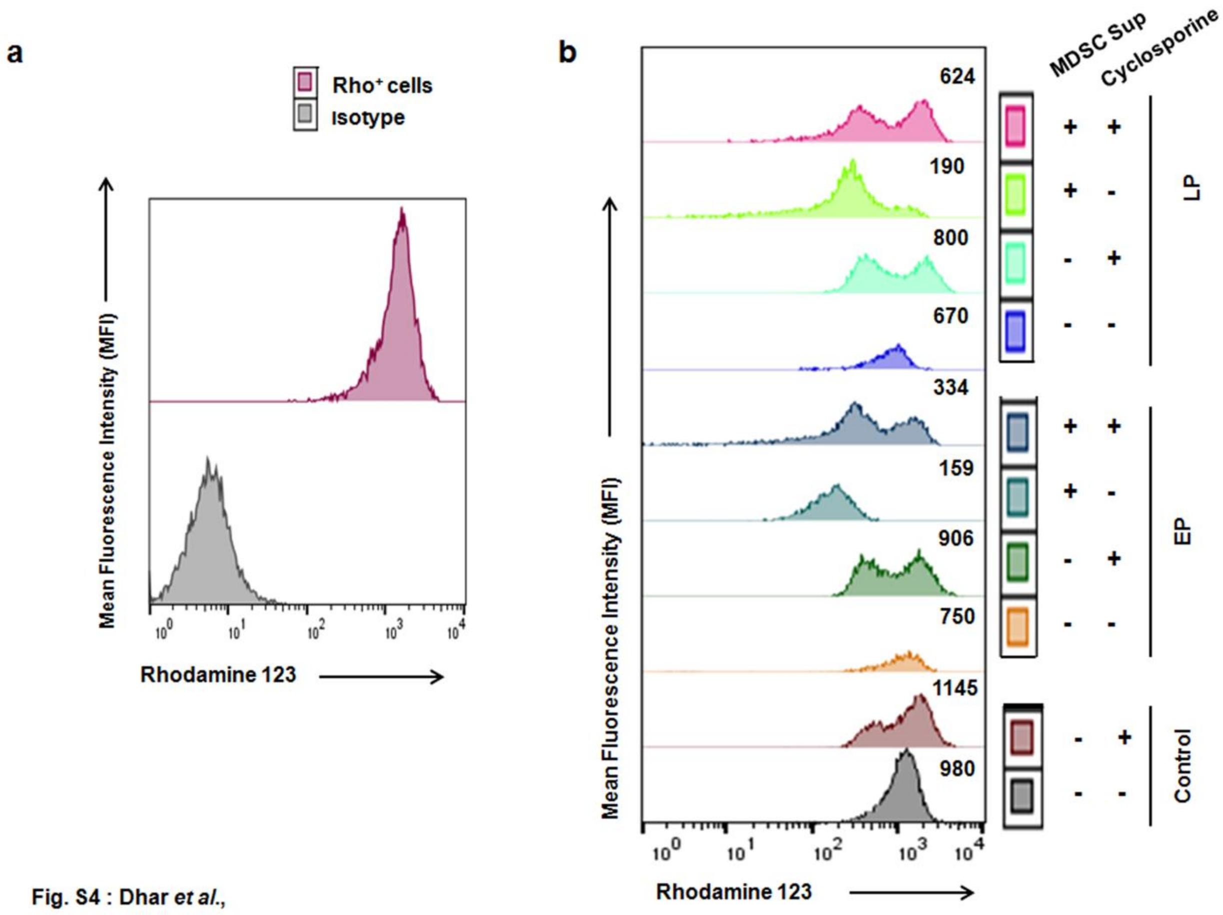


**Supplementary Figure 4: (a)** Representative histogram plot (n= 4) showing the Rhodamine 123$^{+}$ cells in Rhodamine influx assay in comparison to Rhodamine 123$^{-}$ cells. Here cells without Rhodamine dye was considered as unstained sample**. (b)** Histogram plot (n=4) depicting the functional assay of MDR where rhodamine 123 serves as an indicator of cellular influx activity. Representative plot showing the cellular retention of rhodamine dye by mean fluorescence intensity (MFI) in cancer cells (EP, LP) with or without MDSC supernatant and Cyclosporine (MDR blocker).

**Supplementary Figure 5:**

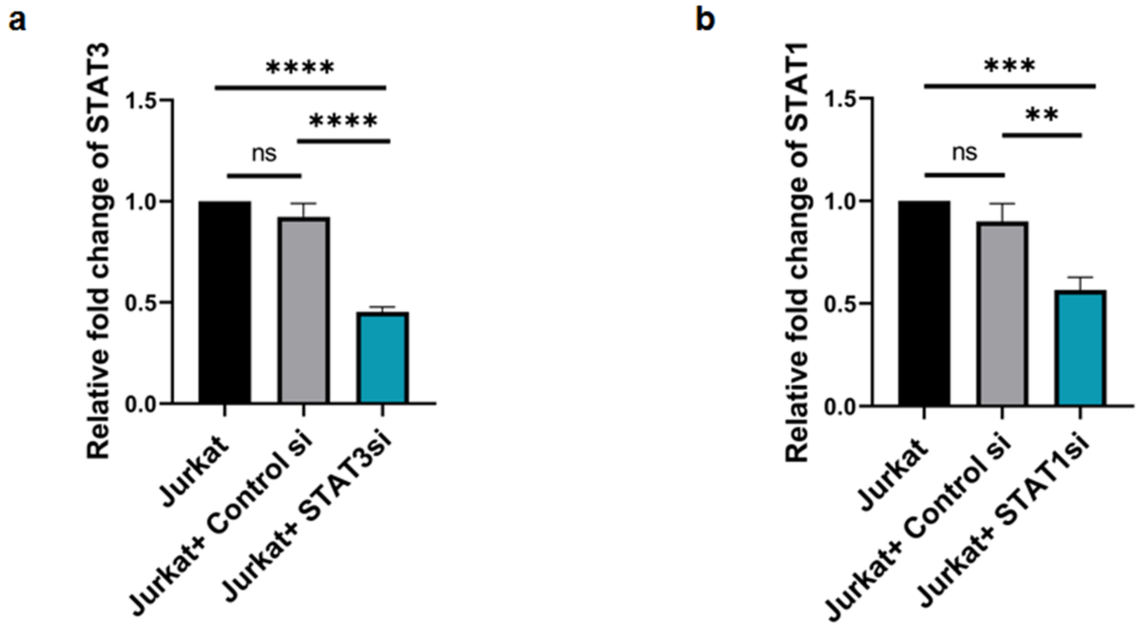


**Fig. S5 : Dhar** ***et al.*****,**

**Supplemenraty Figure 5**: **(a)** Representative bar diagram show the relative fold changes in STAT3 expression in Jurkat cells in presence or absence of STAT3 si-RNA and control si-RNA. **(b)** Representative bar diagram show the relative fold changes in STAT1 expression in Jurkat cells in presence or absence of STAT1 si-RNA and control si-RNA. Bar diagram represent the individual percentage of respective populations with mean±SD in all groups (n=4) for **a**, **b**. Statistical analysis is calculated from One-way ANOVA analysis followed by Tukey's multiple comparison test for both **a**, **b**. **p < 0.05, **p< 0.01, ***p< 0.001, ****p< 0.0001*, ns: not significant are indicated.

**Supplementary Figure 6:**

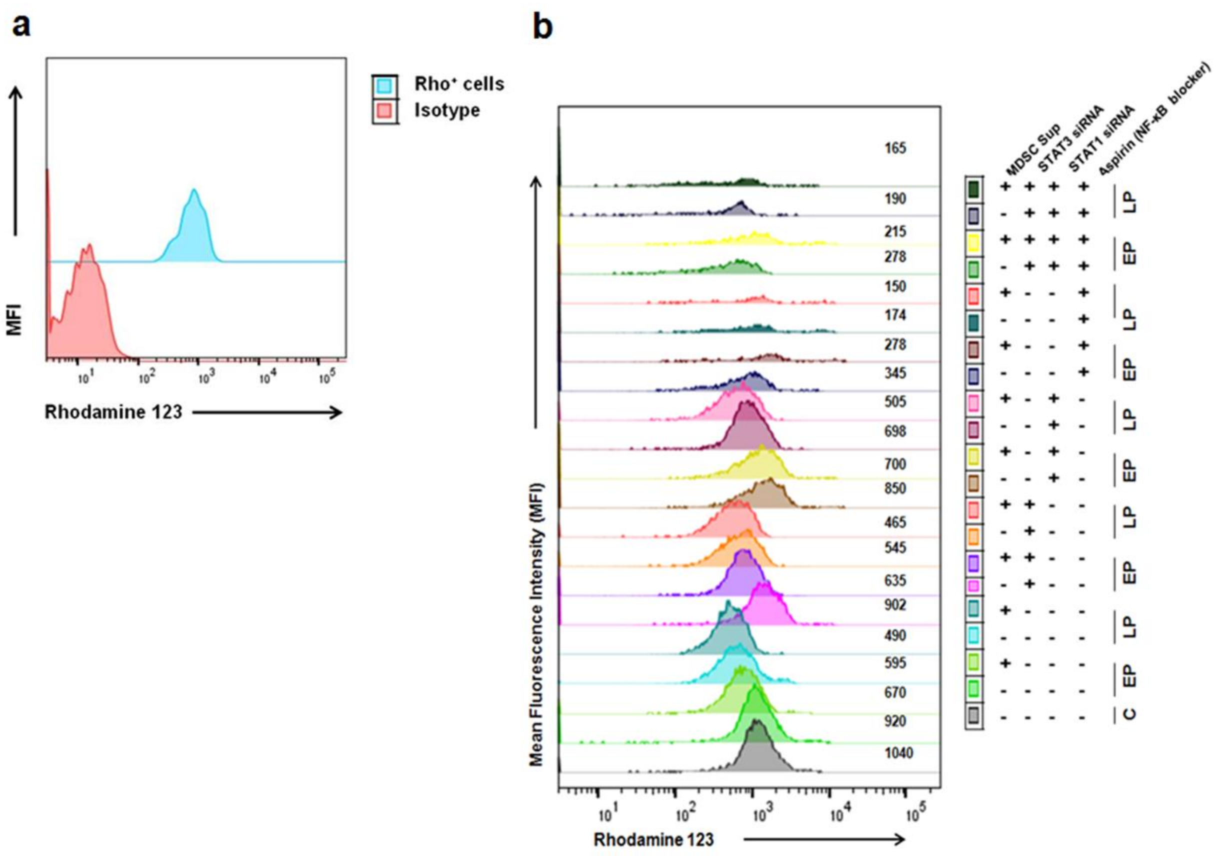


Fig. S6 : Dhar *et al.*,

**Supplementary Figure 6: (a)** Representative histogram plot (n= 4) showing the Rhodamine 123$^{+}$ cells in Rhodamine influx assay in comparison to Rhodamine 123$^{-}$ cells. Here cells without Rhodamine dye was considered as unstained sample**. (b)** Histogram plot (n=3) depicting the functional assay of MDR where rhodamine 123 serves as an indicator of cellular influx activity. Representative plot showing the cellular retention of rhodamine dye by mean fluorescence intensity (MFI) in cancer cells (EP, LP) co-culturing with MDSC supernatant in presence or absence of *stat3* & *stat1* siRNA and NF-κB blocker (Aspirin).
